# Pharmacology, Toxicity, Bioavailability, and Formulation of Magnolol: An Update

**DOI:** 10.3389/fphar.2021.632767

**Published:** 2021-03-17

**Authors:** Yiping Lin, Yuke Li, Yuanlian Zeng, Bin Tian, Xiaolan Qu, Qianghua Yuan, Ying Song

**Affiliations:** ^1^ School of Pharmacy, Chengdu University of Traditional Chinese Medicine, Chengdu, China; ^2^ Affiliated Hospital of Chengdu University of Traditional Chinese Medicine, Chengdu, China

**Keywords:** magnolol, pharmacology, toxicity, bioavailability, formulation

## Abstract

Magnolol (MG) is one of the primary active components of *Magnoliae officinalis* cortex, which has been widely used in traditional Chinese and Japanese herbal medicine and possesses a wide range of pharmacological activities. In recent years, attention has been drawn to this component due to its potential as an anti-inflammatory and antitumor drug. To summarize the new biological and pharmacological data on MG, we screened the literature from January 2011 to October 2020. In this review, we provide an actualization of already known anti-inflammatory, cardiovascular protection, antiangiogenesis, antidiabetes, hypoglycemic, antioxidation, neuroprotection, gastrointestinal protection, and antibacterial activities of MG. Besides, results from studies on antitumor activity are presented. We also summarized the molecular mechanisms, toxicity, bioavailability, and formulations of MG. Therefore, we provide a valid cognition of MG.

## Introduction

**FIGURE 1:**
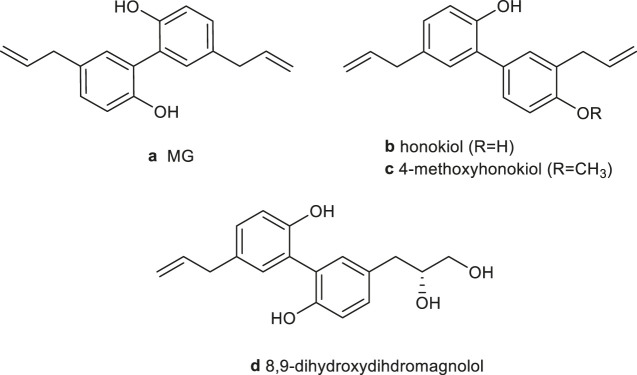
Chemical structure of the main neolignans of *Magnoliae officinalis* cortex.

*Magnoliae officinalis* cortex, which was first recorded in “Shennong Herbal Classic” (Qin and Han Dynasty, around 221 B.C. to 220 A.D.), is the dry bark, root bark, and branch bark of *Magnolia officinalis* Rehd. *et* Wils. or *Magnolia officinalis* Rehd. *et* Wils. var. *biloba* Rehd. *et* Wils. In traditional medicine, *Magnoliae officinalis* cortex mainly acts to dry dampness and disperse phlegm, lower Qi, and eliminate fullness. Clinically, it is commonly used to treat asthma, constipation, edema, abdominal distension, malaria, and other diseases by combining different traditional Chinese medicines. For example, the Da Houpo Pill is used to treat abdominal distension (Song Ji Zonglu). The Xiaochengqi decoction is used for the treatment of tidal fever, constipation, and abdominal pain (Treatise on Febrile Diseases). The Banxia Houpo decoction has therapeutic effects on chronic pharyngitis, chronic bronchitis, and esophageal fistula (Synopsis of the Golden Chamber). Recent studies have shown that *Magnoliae officinalis* cortex has multiple pharmacological activities on the nervous system (Lee et al., 2009; Lee et al., 2013), digestive system (Kim HJ et al., 2018), inflammation (Kim JY et al., 2018), and cancer (Kim et al., 2020). And, its neolignan compounds include MG (a), honokiol (b), 4-methylhonokiol (c), and (R)-8,9-dihydroxydihydromagnolol (d) (Rempel et al., 2013) (Figure 1).

The isomers MG (5,5′-diallyl-2,2′-dihydroxybiphenyl) and honokiol (3,5′- diallyl-4,2′-dihydroxybiphenyl) are biphenyl-type neolignans. They have been recognized as the principal active components of magnolia bark extract, usually accounted for 1–10% of dry bark, depending on the *Magnolia* species (*officinalis* or *obovata*) and extraction method (Sarrica et al., 2018; Oufensou et al., 2019; Łata et al., 2020). Talarek *et al.* reviewed the chemistry, bioavailability, and neuroprotective activity of honokiol (Talarek et al., 2017). Woodbury *et al.* concluded that honokiol has therapeutic potential for anxiety, pain, cerebrovascular damage, epilepsy, and cognitive disorders (Woodbury et al., 2013). Ong *et al*. and Banik *et al.* summarized the antitumor mechanisms of honokiol, including the regulation of MAPK, NF-κB, HIF-α, PI3K/Akt/ERK/mTOR, Wnt/β-catenin epidermal growth factor receptor (EGFR), signal transduction and activator of transcription (STAF), and notch signaling pathways (Rauf et al., 2018; Banik et al., 2019; Ong et al., 2020). The metabolism, bioavailability, and pharmacological of honokiol were reviewed by Ong *et al.* (Ong et al., 2020). Additionally, the antiangiogenesis (Fried and Arbiser, 2009), antioxidation and antibacterial activities (Shen et al., 2010), and molecular mechanisms of honokiol have been summarized.

MG was first isolated from magnolia bark by Japanese scientist Sugii in 1930 and was first synthesized by Swedish scientist H. Erdtman and J. Runebeng with the p-allylphenol as raw material (Erdtman and Runeberg, 1957). However, the yield was only 25%, and it was challenging to separate and purify. Zhang *et al.* used 2,2′-biphenol and 1-bromobutane as raw materials to prepare MG (Zhang and Sun, 2011). The reaction process was simple and effective with mild conditions as well as high product purity (>98%), and the yield was increased to 60.2%.

Numerous studies showed that MG possesses extensive biological activities, such as anti-inflammatory (Wei et al., 2014; Lin et al., 2015; Zhang L et al., 2018; Chen H et al., 2019), antitumor (McKeown et al., 2014; Zhang FH et al., 2017; Shen et al., 2017), cardiovascular protection (Liang CJ et al., 2014; Chang et al., 2018), antiangiogenesis (Kim GD et al., 2013; Chen et al., 2013), hypoglycemic (Pulvirenti et al., 2017; Suh et al., 2017; Parray et al., 2018), antioxidation (Baschieri et al., 2017), neuroprotection (Matsui et al., 2016; Kou et al., 2017; Xie et al., 2020), gastrointestinal protection (Chao et al., 2018), and antibacterial activities (Dong et al., 2017) (Table 1).

**TABLE 1:** Modern pharmacological studies of MG.

Effect	Model/targets		Positive		Dosage	Result/mechanism/method	References
Anti-inflammatory activity	LPS-induced RAW 264.7 cells				*In vitro*: 5, 10, and 15 μM	Inhibited iNOS and COX-2 expression and NF-κB activation via regulating PI3K/Akt and MAPK signaling pathways	Lai et al. (2011)
	MTT-induced U937 cells				*In vitro*: 10–100 μM	Inhibited NO production and expression of p-IκBα, p-P65, IL-1β, and TNF-α. Downregulated phospho-JNK (p-JNK) and p-p38	Chen H et al. (2019)
	C57BL/6 mice		DXM (5 mg kg^−1^) increased colon length and relieved colon pathological injuries		*In vivo*: 5, 10, and 20 mg kg^−1^ (intragastric administration)	Dose-dependently reduced TNF-α, IL-1β, and IL-6. Inhibited weight loss and colon shortening induced by dextran sulfate sodium (DSS)	
	DSS-induced male C57BL/6 mice				*In vivo*: 25, 50, and 100 mg kg^−1^ (gavage)	Inhibited the expression of TNF-α, IL-1β, and IL-12 by regulating NF-κB and PPAR-γ pathways	Shen P et al. (2018)
	RAW 264.7 cells				*In vitro*: 5, 10, and 20 μM	Activated p38 MAPK and Nrf2/HO-1 cascade and promoted ROS production	Lu et al. (2015b)
	LPS-induced mammary tissues				*In vitro*: 12.5, 25, 50, 100, and 200 μg ml^−1^	Reduced phosphorylation of p65, p38, IκBα, JNK, and ERK. Inhibited TLR4 expression and production of TNF-α, IL-1β, and IL-6	Wei et al. (2014)
	LPS-induced mouse uterine epithelial cells				*In vitro*: 12.5, 25, and 50 μg ml^-1^	Inhibited the expression of TLR4 and NF-κB and MAPKs activation	Luo et al. (2013)
	LPS-induced BALB/c mice		DEX (0.5 mg kg^−1^) reduced the MPO activity		*In vivo*: 5, 10, and 20 mg kg^−1^ (i.p. injection)	Attenuated mice mastitis tissue damage and MPO activity	Wei et al. (2014)
	LPS-induced SD rats				*In vivo*: 10 and 20 mg kg^−1^ (i.p. injection)	Increased the expression of PPAR-γ. Altered pneumonedema, neutrophil infiltration, ROS production, iNOS and COX-2 expression, NF-κB activation, and proinflammatory factor level	Lin et al. (2015)
	LPS-induced RAW 264.7 cells				*In vitro*: 15, 30, and 60 μg ml^−1^	Downregulated TLR4 expression, NF-κB and MAPK pathway activation, and proinflammatory cytokine excretion. Dose-dependently (30–60 μg ml^−1^) inhibited the IL-1β, IL-6, and TNF-α expression. Suppressed IκBα degradation and phosphorylation of JNK, ERK, and p38	Fu et al. (2013)
	Human FLS				*In vitro*: 2.5–25 μg ml^−1^	Suppressed cytokine expression and MAPKs and IκB/IκB kinases/NF-κB pathway in a dose-dependent manner	Wang et al. (2012)
	Female Lewis rats				*In vivo*: 100 mg kg^−1^ (i.p. injection)	Attenuated paw swelling and serum cytokine levels	
	C57BL/6J mice				*In vivo*: 10, 25, and 50 mg kg^−1^ (i.p.)	Decreased the expression of inflammatory cytokines and inhibited HIF-1α/VEGF pathway	Yang et al. (2016)
	RAW 264.7 cells				*In vitro*: 25, 50, and 100 μM	Declined the production of inflammatory cytokines and ROS and the expression of TLR2. Prevented p38, ERK, JNK, and NF-κB phosphorylation	Zhang P et al. (2017)
	A549 cells				*In vitro*: 6.25, 12.5, 25, 50, 100, and 200 μM	Suppressed NF-κB and MAPK pathway activation by reducing the upregulation of intercellular adhesion molecule-1 and phosphorylation of NF-κB, p38, ERK1/2, and SAPK/JNK	Wu et al. (2014)
	Human aortic endothelial cells				*In vitro*: 5 μM	Reduced leukocyte adhesion via inhibiting JNK/P38 phosphorylation, NF-κB activation, and HuR translocation	Liang CJ et al. (2014)
Antitumor activity	Cholangiocarcinoma (CCA) cells		5-FU, CDDP, and GEM (40 μM) reduced cell survival		*In vitro*: 20–160 μM	Suppressed the growth, migration, and invasion of CCA cells by regulating cell cycle and expression of cyclin D1 protein, PCNA, Ki67, MMP-2, MMP-7, and MMP-9	Zhang FH et al. (2017)
	BALB/c nude mice				*In vivo*: 40 mg kg^−1^ (i.p. injection)	Reduced the growth and weight of tumor	Zhang FH et al. (2017)
	SKOV3 human ovarian and BT474 human breast cancer cells				*In vitro*: 6.25, 12.5, 25, 50, 100, and 200 μΜ	Inhibited the overexpression of HER2 gene by decreasing PI3K/Akt and inhibiting the expression of VEGF, MMP2, and cyclin D1	Chuang et al. (2011)
	Human non-small-cell lung cancer cell lines				*In vitro*: 1, 5, 10, and 20 μM	Inhibited NCI-1299 and A549 cells (IC_50_ = 5 µM) by blocking cell cycle, destroying cellular microtubule tissue, reducing Akt/mTOR pathway, and promoting autophagy	Shen et al. (2017)
	Male nude mice				*In vivo*: 25 mg kg^−1^ (i.p. injection)	Significantly reduced tumor size and weight	
	Human HCT116, SW480, and HEK293 cells				*In vitro*: 12.5, 20, 25, 30, 50, and 75 μM	Regulated the Wnt/β-catenin signaling pathway and β-catenin/T-cell factor-targeted downstream genes. Inhibited tumor cell invasion and motility	Kang et al. (2012)
	Female nude mice				*In vivo*: 5 mg kg^−1^ (i.p. injection)	Inhibited the tumor growth effectively with an inhibition rate of 54.6%	
	Human gastric adenocarcinoma SGC-7901 cells				*In vitro*: 10, 30, 50, 100, 200, and 300 µM	Regulated the mitochondria and PI3K/Akt-dependent pathways, Bax/Bcl-2 ratio, caspase-3 activation, PI3K/Akt inhibition, and cell apoptosis and induced autophagy	Rasul et al. (2012)
	GBC cell lines				*In vitro*: 10, 20, and 30 μM	Altered levels of p53, p21, cyclin D1, CDC25A, and Cdk2, blocked cell cycle progression, and induced mitochondria-related apoptosis	Li et al. (2015)
	BALB/c homozygous nude mice				*In vivo*: 5, 10, 20, 30, 40, and 50 mg kg^−1^ (i.p. injected)	Suppressed the tumor growth and CDC2 expression and increased caspase-3 activation	Li et al. (2015)
	Human DU145 and PC3 prostate adenocarcinoma cells				*In vitro*: 40 and 80 μM	Modulated the cell cycle process of PC3 and DU145 cells. Downregulated the expression of A, B1, D1, E, CDK2, CDK4, and pRBp130. And increased pRBp107 protein expression level	McKeown et al. (2014)
	Human PC3 cells and LNCaP cells				*In vitro*: 80 μM	Affected the expression of insulin-like growth factor-1 (IGF-1), and associated proteins including IGF-binding protein-5 (IGFBP-5), IGFBP-3, IGF-1 receptor, and IGFBP-4	McKeown and Hurta (2014)
	HCT-116 cells				*In vitro*: 1, 5, 10, 25, and 50 μM	Promoted cell apoptosis and inhibited migration and invasion of HPT-116 cells by decreasing Bcl-2 expression, increasing the expression of p53 and Bax, and activating AMPK and caspase-3	Park et al. (2012)
	Human lung carcinoma A549 cells				*In vitro*: 1, 5, 10, 50, and 100 µM	Upregulated the release of lactate dehydrogenase, facilitated caspase-3 activation and poly-(ADP)-ribose polymerases cleavage, and reduced NF-κB/RelA expression level. Inhibited A549 cells growth	Seo et al. (2011)
	Nude immunodeficient mice				*In vitro*: 40 mg kg^−1^ (i.p. injection)	Markedly inhibited the growth of MDA-MB-231 and McF-7 tumors and MMP-9 level	Liu et al. (2013)
	Human breast cancer cell lines and nontumorigenic MCF-10A mammary epithelial cells				*In vitro*: 10, 20, 30, 40, 50, and 60 µM	Prevented breast cancer cell invasion via inhibiting NF-κB pathway and MMP-9 expression	Liu et al. (2013)
	WM1366 (NRAS-mutated) and WM164 (BRAF-mutated) cell lines				*In vitro*: 10, 20, and 30 µM	Inhibited BRAF/MEK and induced cell death by significantly downregulating PI3K/Akt pathway	Emran et al. (2019)
	PC3 cells				*In vitro*: MG 80 μM	Decreased the protein expression of ornithine decarboxylase, R2 subunit of ribonucleotide reductase, p-p38, JNK-1/2, PI3Kp85, p-PI3Kp85, p-Akt, NFκBp65, p-IκBα, and IκBα. And increased the protein expressions of p-JNK-1, and c-Jun	McKeown and Hurta (2015)
	MCF7 cells				*In vitro*: 20 µM	Increased the expression of the tumor suppressor miRNA miR-200c	Hagiwara et al. (2015)
Antiangiogenic activity	T24 and HUVEC cells				*In vitro*: 1, 5, and 10 μM	Inhibited HIF-1α/VEGF-dependent pathways, H_2_O_2_ formation, mRNA and protein expression, and transcriptional and VEGF excretion	Chen et al. (2013)
	Female athymic nude mice (BALB/c)				*In vivo*: 2, 5, and 10 mg kg^−1^ (i.p. injection)	Decreased angiogenesis, HIF-1A, VEGF, CD31, and carbonic anhydrase-IX expression	Chen et al. (2013)
	MES/EB-derived endothelial-like cells				*In vitro*: 5, 6.25, 10, 12.5, 2 0, 25, 50, and 100 μM	Inhibited PECAM transcription, translational expression, and MAPKs/PI3K/AKT/mTOR signaling pathway activation	Kim GD et al. (2013)
	HUVEC cells				*In vitro*: 10 and 40 μM	Suppressed proliferation, ERK1/2 activity, gelatinase activity, and ROS production and promoted HO-1 level	Kuk et al. (2017)
	Male NMRI mice				*In vivo*: 20 μg/ear (transdermally administered)	Inhibited venous remodeling process and decreased endothelial proliferation and MMP-2 abundance. Amplified HO-1-mediated resistance of endothelial cells to ROS-mediated proliferative stimuli	Kuk et al. (2017)
Cardiovascular protection	Right coronary arteries from hearts of pigs				*In vitro*: 1, 3, 10, 30, and 100 μM	Relaxed the coronary artery with an IC_50_ value of 5.78 μM and dose-dependently inhibited iNOS and COX-2 protein expression	Kuo et al. (2011)
	Human aortic smooth muscle cells (HASMCs)				*In vitro*: 10, 20, and 30 μM	Inhibited VSMC migration by suppressing cytoskeletal remodeling and neointima formation	Karki et al. (2013b)
	Male SD rats				*In vivo*: 1, 10, and 100 μg kg^−1^ (intravenous (i.v.) injection)	Reduced the proportion of myocardial ischemic necrosis area. At a concentration of 10 μg kg^−1^, MG reduced ventricular fibrillation and animal mortality	
	Male SD rats		Ticlopidine 100 mg kg^−1^ decreased intimal area as well as intimal/medial ratio and increased luminal area		*In vivo*: 50 and 100 mg kg^−1^ (gavage)	Attenuated neointima formation, intimal area, and intimal/medial ratio and increased luminal area	Karki et al. (2013a)
	Male SD rats				*In vivo*: 10 mg kg^−1^ (i.p.)	Regulated ACE/Ang II/AT-1R cascade and ACE2. Attenuated the overexpressions of ET-1 and ETA receptor by suppressing Akt/ERK1/2/GSK3β-catenin pathway	Chang et al. (2018)
	Male spontaneous hypertensive rats				*In vivo*: 100 mg kg^−1^ (gavage)	Decreased blood pressure through upregulating PPAR-γ, Akt, and eNOS activity, downregulating TRB3, and improving vascular insulin resistance	Liang X et al. (2014)
	VSMCs				*In vitro*: 5, 10, and 20 μM	Suppressed VSMC proliferation and DNA synthesis by inhibiting the expressions of cyclin D1/E, cyclin-dependent kinase 2 and 4, ROS production, and activation of renin–angiotensin system, MEK, and ERK1/2	Wu et al. (2015)
Hypoglycemic activity	3T3-L1 and HIB1 B preadipocytes				*In vitro*: 1, 5, 10, and 20 μM	Enhanced adipocyte differentiation and expression of brown adipocyte-specific marker genes and proteins. Promoted browning of 3T3-L1 fat cells via activating AMPK, PPAR, and PKA pathways	Parray et al. (2018)
	Protein tyrosine phosphatase-1B (PTP1B)				*In vitro*: 5, 10, 20, and 30 μM	Inhibited PTP1B in dose-dependent manner with an IC_50_ value of 24.6 μM	Onoda et al. (2016)
	RIN-m5F cells				*In vitro*: 0.01, 0.1, and 1 μM	Increased insulin secretion, Ins2 and PDX1 expression, and levels of AMPK phosphorylation, SIRT1, and PGC1α. Prevented protein glycation	Suh et al. (2017)
	α-Glucosidase				*In vitro*: 0.5–100 μM	Inhibited α-glucosidase with an IC_50_ value of 2.0 μM and 29.8% inhibition at a concentration of 1.5 μM	Pulvirenti et al. (2017)
	L6 rat myoblast				*In vitro*: 3, 10, and 30 μM	Promoted glucose uptake in a dose-dependent manner and stimulated glucose transporter-4 translocation to the cell surface via enhancing Akt phosphorylation	Choi et al. (2012)
	Insulin-sensitive/resistant murine and human adipocytes				*In vitro*: 0.1, 1, 10, and 30 μM	Promoted glucose uptake by regulating insulin signaling pathway	Alonso-Castro et al. (2011)
					
Gastrointestinal protection	Castor oil-induced male Kunming mice		Saline (20 mg kg^−1^) relieved diarrhea		*In vivo*: 25, 50, and 100 mg kg^−1^ (gavage)	Inhibited diarrhea in mice significantly. Reduced neostigmine-induced small intestinal transit, and increased activity of CAT, SOD, and GSH-Px	Pang et al. (2013)	
	ETEC-induced diarrhea male Kunming mice				*In vivo*: 100, 300, and 500 mg kg^−1^ (gavage)	Regulated the release of IP3-Ca^2+^ storage, suppressed SK channel, and facilitated the opening of BKα1 and BKβ3 channels and the closing of BKβ4 channel	Deng et al. (2015)	
	Colonic smooth muscle cells from male SD rats				*In vitro*: 1, 3, 10, 30, and 100 μM	Downregulated L-type Ca^2+^ channel activity to inhibit the spontaneous contractions of colonic smooth muscle occur in a concentration-dependent manner	Zhang et al. (2013)	
	Kunming mice				*In vivo*: 5, 10, 15, 20, 25, 30, and 40 mg kg^−1^ (gavage)	It had significant inhibitory effects on the small intestine charcoal propulsion induced by rhubarb, diarrhea induced by Senna leaf, and gastric emptying inhibition induced by atropine	Zeng et al. (2015)	
Neuroprotection	CMS-induced male Kunming mice		Fluoxetine (20 mg kg^−1^) increased sucrose preference		*In vivo*: 20 and 40 mg kg^−1^ (gavage)	Inhibited prefrontal cortex oxidative stress and depression-like behavior by reducing the levels of IL-1β, IL-6, and TNF-α, microglia cell activation, HPA axis hyperactivity and lipid peroxidation, and increasing enzymes decrease	Cheng et al. (2018)	
	CMS-induced male ICR mice		Fluoxetine (20 mg kg^−1^) decreased immobility duration and serum CORT levels	*In vivo*: 50 and 100 mg kg^−1^ (gavage)		Modulated HPA axis and upregulated BDNF protein, 5-HT, and norepinephrine (NE) level. Decreased CORT level	Bai et al. (2018)	
	Olfactory bulbectomy male ddY mice		Fluoxetine (20 mg kg^−1^) ameliorated the depression-like behavior	*In vivo*: 50 and 100 mg kg^−1^ (gavage)		Ameliorated depression-like behavior and hippocampal nerve injury significantly	Matsui et al. (2016)	
	Male SD rats subjected to unpredictable CMS (UCMS)		Fluoxetine hydrochloride (20 mg kg^−1^) reversed depression-like behavior	*In vivo*: 20 and 40 mg kg^−1^ (gavage)		Ameliorated depression-like behaviors via reversing glial atrophy	Li LF et al. (2013)	
	UCMS-induced male SD rats		Fluoxetine hydrochloride (20 mg kg^−1^) increased the levels of 5-hydroxyindoleacetic acid and 5-HT	*In vivo*: 20 and 40 mg kg^−1^ (gavage)		Increased BDNF expression and serotonergic system activity	Li et al. (2012)	
	Male Kunming strain mice		Diazepam (2 mg kg^−1^) prolonged the latency of epileptic seizures and increased the latency of myoclonic Jerks	*In vivo*: 20, 40, and 80 mg kg^−1^ (i.p. injection)		Delayed myoclonic jerks and myoclonic seizures. Declined seizure stage and mortality by affecting GABAA/benzodiazepine receptor	Chen CR et al. (2011)	
	BV2 cells			*In vitro*: 2.5, 5, 10, 50, 100, 150, and 200 μM		Increased Aβ phagocytosis and degradation and ApoE level by activating the target gene liver-X-receptor of PPAR-γ	Xie et al. (2020)	
	Male SD rats			*In vivo*: 30 mg kg^−1^ (i.p. injection)		Attenuated brain water content and neurological deficits and restored the BBB by reducing glial cell stimulation, neutrophil infiltration, and production of IL-1β, TNF-α, and MMP-9	Zhou F et al. (2019)	
	TMT-induced HT22 cells and BV-2 cells			*In vitro*: 5, 10, 15, and 20 μM		Inhibited neuronal cell death and microglial activation by suppressing ROS production and activation of JNK, p38 MAPKs, and NF-κB	Kim and Kim (2016)	
	TMT-induced male ICR mice			*In vivo*: 25 mg kg^−1^ (i.p. injection)		Reversed a large number of neuronal injury and oxidative stress induced by TMT. Decreased glial cells and iNOS expression and blocked the activation of JNK and P38				
	Glutamate-induced neurons			*In vitro*: 0.1, 1 μM		Attenuated intracellular Ca^2+^ levels, [Ca^2+^]i increase, cytotoxicity, and cell swelling	Lee et al. (2012)	
	Male SD rats			*In vivo*: 25, 50, 100, 150, and 200 mg kg^−1^ (i.p.)		The infarct area was significantly reduced by 30.9–37.8%, and neurobehavioral scores were improved	Lee et al. (2012)	
	Stroke male SD rats			*In vivo*: 10 and 30 mg kg^−1^ (i.p. injection)		Deduced the levels of IL-1β, IL-6, and TNF-α. Inhibited the production of 4-HNE, iNOS, nitrotyrosine, C/EBP homologs, and phosphorylated p38MAPKs	Chen et al. (2014)	
	LPS-induced microglial cells, brain microvascular endothelial cells			*In vitro*: 0.01, 0.1, 1, and 10 μM		Attenuated the BBB hyperpermeability in a dose- and time-dependent manner. Reduced levels of iNOS, TNF-α, and IL-1β and p65 subunit expression	Liu et al. (2017)	
	I-R-induced Kunming mice		Edaravone (3 mg kg^−1^) reduced about 33% of the white infarct areas and failed to inhibit Evans blue secretion and brain edema	*In vivo*: 1.4, 7.0, and 35.0 μg kg^−1^ (i.v. injection)		Reduced infarct volume, cerebral water content, and Evans blue secretion				
	Fluid percussion-induced male SD rats			*In vivo*: 0.2 and 2 mg kg^−1^ (i.v. injection)		Reduced cerebral infarction volume and neuronal apoptosis. Increased the expression of transforming growth factor-β1	Wang CC et al. (2013)	
	Neuronal NG108-15 cells			*In vitro*: 10, 30, and 100 μM		Inhibited the voltage K^+^ and voltage-gated Na^+^ channels with IC50 values of 21 and 15–30 μM	Gong et al. (2012)	
Interaction with CYP450 enzyme	Male SD rats			*In vivo*: 50 mg kg^−1^ (gavage)		Inhibited CYP1A and 2C significantly	Duan et al. (2015)	
	Rat/human CYP enzymes (1A2/1A2, 2D/2D6, 3A/3A4, 2E1/2E1, and 2C/2C9)			*In vitro*: 8, 10, 16, 32, and 64 μM		Inhibited human CYP3A_4_ and rat CYP1A2 with IC_50_ values of 56.2 and 10 μM, respectively	Duan et al. (2015)	
	Human CYP2C19		The IC_50_ value of 1.37 μM for loratadine	*In vitro*: 0.1, 0.25, 0.5, 0.75, 1, 1.5, and 2 μM		Noncompetitive inhibition of CYP2C19 with IC_50_ and Ki values of 1.37 μΜ and 10.0 μM, respectively	Zhang T et al. (2018)	
	SD rat CYP2D			*In vitro*: 3.91–125 μM		Inhibited the CYP2D in a dose-dependent manner, with an IC_50_ value of 39.9 μM. And inhibited rat CYP2C8, CYP2E1, and CYP2A1/2 and human CYP2E1 and CYP2A6 with IC_50_ values > 100 μM	Liu et al. (2016)	
	CYP2C, CYP2D6, CYP2E1, CYP3A4, CYP1A2, and CYP2B6			*In vitro*		The IC_50_ values of MG on rat CYP2C, CYP2D6, CYP2E1, CYP3A4, CYP1A2, and CYP2B6, were 5.56 ± 2.87 μM, 65.42 ± 4.46, 67.93 ± 9.51, 52.36 ± 17.32, 97.80 ± 3.83, and 28.69 ± 1.46, respectively	Huang et al. (2019)	
	Male SD rats			*In vivo*: 5 mg kg^−1^		The mean IC_50_ values of MG for the metabolism of phenacetin and diclofenac were 19.0 and 47.3 µM, respectively	Kim SB et al. (2018)	
	CYP1A2, CYP2B6, CYP2C8, CYP2C9, CYP2C19, CYP2D6, and CYP3A			*In vitro*: 0.5–50 µM		The IC_50_ values for the CYP1A2, CYP2B6, and CYP2C9 were 5.4, 44.9, and 10.2, respectively	Joo and Liu (2013)	
Antibacterial activity	*Aeromonas hydrophila* strains			*In vitro*: 2, 4, 6, 8, and 16 μg ml^−1^		The MIC values ranged from 32–64 μg ml^−1^	Dong et al. (2017)	
	MRSA, MSSA, and ATCC 25923			*In vitro*: 8–128 mg L^−1^		The MIC50/MBC50 values of MSSA and MRSA were 32/32 and 16/16 mg L^−1^, respectively	Zuo et al. (2015)	
	64 *Candida* spp. strains		Amphotericin B (the range of MIC value was 0.12–0.5 μg ml^−1^)	*In vitro*: 0.5–256 μg ml^−1^		The range of MIC value was 16–64 μg ml^−1^	Behbehani et al. (2017)	
	32 *Fusarium* spp. strains			*In vitro*: 5–400 μg ml^−1^		MG had similar bactericidal activity compared with fluconazole; however, compared with terbinafine, it was less effective at all selected concentrations	Oufensou et al. (2019)	
	*A. actinomycetemcomitans, S. mutans, S. aureus,* MRSA, and *E. coli*		Cycloheximide, the MIC/MBC values of <1/<1, <1/<1, 1</2, 1</<1, and 1</<1 μg ml^−1^	*In vitro*: 1–100 μg ml^−1^		The MIC/MBC values were 10/20, 10/20, 10/30, 20/90, and >100/>100 μg ml^−1^, respectively	Chiu et al. (2020)	
	*Alternaria alternata* (Fr.) Keissl, *Penicillium expansum* (Link) Thom, *Alternaria dauci* f.sp. solani, *Fusarium moniliforme* J. Sheld, *Fusarium oxysporum* Schltdl., *Valsa mali* Miyabe & G. Yamada, and *Rhizoctonia solani* J.G. Kühn A			*In vitro:* 0.001, 0.005, 0.01, 1, 3, 5, and 7 mg ml^−1^		The growth inhibition rate of 7 pathogenic fungi was over 57%	Chen Y-H et al. (2019)	
Antioxidative activity	Acrolein-induced SH-SY5Y human neuroblastoma cells			*In vitro*: 8, 16, and 32 μM		Played roles in protecting against oxidative stress and prolonging the vitality in acrolein-induced SH-SY5Y cells by altering JNK/mitochondria/caspase, PI3K/MEK/ERK/Akt/O subfamily of FoxO 1 signaling pathways	Dong et al. (2013)	
	AA-induced HK-2 cells			*In vitro*: 5 and 10 μM		Effectively reduced oxidative stress, suppressed cell proliferation, and prevented the G2/M arrest induced by AA.	Bunel et al. (2016)	
	Male C3H/HeOuJ mice		Hypertonic saline (4 ml kg^−1^ 7.5%) reduced interstitial edema and blood DHR 123 oxidation	*In vivo*: 20 mg g^−1^ (i.v. injection)		Attenuated lung injury by significantly reducing pulmonary edema, iNOS expression, MPO activity, and plasma peroxynitrite	Shih et al. (2012)	
Antiphotoaging activity	UVB-induced HR-1 hairless male mice			*In vitro*: 40 μL of the formulation containing 0.25% MG (topically applied)		Reduced the mean length and depth of wrinkles and levels of MMP-1, MMP-9, and MMP-13	Im et al. (2015)	
Inhibition of osteoclast differentiation	RAW 264.7 macrophages			*In vitro*: 2.5, 5, 10, and 20 μM		Suppressed MAPK/c-fos/AP-1/NF-κB signaling and ROS production. Increased HO-1 expression	Lu et al. (2015a)	
	TDSCs			*In vitro*: 5, 10, or 20 μM		Inhibited ALP activity and calcium deposits	Zhou W et al. (2019)	
	Male SD rats			*In vivo*: 20 mg kg^−1^ (i.p. injection)		Suppressed the expressions of RUNX2, OCN, and BMP2		
	MC3T3-E1 cells			*In vitro*: 0.01, 0.1, and 1 μM		Significantly downregulated the production of osteoclast differentiation-inducing factors such as RANKL, TNF-α, and IL-6 and inhibited mitochondrial electron transport	Kwak et al. (2012)	
	RANKL-induced RAW 264.7 macrophages			*In vitro*: 75, 100, and 150 μM		Decreased osteoclast differentiation, tartrate-resistant acid phosphatase activity of differentiated cells, and resorption pit area caused by osteoclasts in a concentration-dependent manner	Lu et al. (2013)	
	Male SD rats			*In vivo*: 100 mg kg^−1^ (p.o.)		Significantly suppressed alveolar bone resorption, the number of osteoclasts on the bony surface, expression of RANKL, MMP-1, MMP-9, iNOS and COX-2, and TNF-α activation	Lu et al. (2013)	
	Primary osteoblasts			*In vitro*: 1.25, 5, and 10 μM		Inhibited IL-1-induced RANKL expression and osteoclast differentiation by suppressing COX-2 expression and PGE2 production	Hwang et al. (2018)	
Antiparasitic activity	Ichthyophthirius multifiliis	Malachite green (0.05 mg L^−1^) inhibited existence		*In vitro*:0.2, 0.3, 0.4, 0.5, 0.6, 0.7, and 0.8 mg L^−1^		When treated theronts with 0.6 mg L^−1^ or higher concentration of MG for 4 h, the fatality rate was 100%	Song et al. (2018)	
	Infected fish	Malachite green (0.25 and 0.5 mg L^−1^) reduced theronts release		*In vivo*: 1.5, 2.5, and 3.5 mg L^−1^		Markedly decreased the quantity of theronts release	Song et al. (2018)	
Antiviral activity	Grass carp reovirus infection in CIK cells			*In vitro*: 1.5 μg ml^−1^		Facilitated the expression of type I interferon regulatory factor to inhibit grass carp reovirus	Chen et al. (2017)	
	HBV-transfected HepG2.2.15 cell line					Inhibited HBV activities significantly with IC_50_ values of 2.03, 3.76, and 8.67 μM for HBsAg, HBeAg, and replication of HBV DNA, respectively	Li J et al. (2013)	
Reduction of multidrug resistance	NCI/ADR-RES cells			*In vitro*: 1, 5, 10, 25, and 50 μM		Reduced the multidrug resistance of cancer cells to antitumor drugs by downregulating P-glycoprotein expression in a concentration- and time-dependent manner	Han and Van Anh (2012)	

The studies about MG’s toxicity have been done, suggesting that MG has no genotoxicity and mutagenic toxicity (Saito et al., 2006). As a phenolic polyhydroxy compound, MG’s poor aqueous solubility and low oral bioavailability limit its clinical use. Therefore, various formulations such as liposomes (Shen et al., 2016), solid dispersions (Stefanache et al., 2017a), emulsions (Sheng et al., 2014), and nanoparticles (Wang et al., 2011) have been developed to ameliorate the water solubility and bioavailability of it.

In this review, the pharmacological activities and molecular mechanisms of MG are summarized and updated. Its toxicities, bioavailability, and formulations are reviewed, to identify the benefit of further studies on MG and to find the best method to improve its bioavailability.

## Materials and Methods

This article collected literature studies related to pharmacology, toxicity, bioavailability, and formulation of MG published from January 2011 to October 2020. All related information about MG was collected by using the keyword of magnolol from globally recognized scientific search engines and databases, such as Web of Science, Springer, ScienceDirect, Elsevier, Google Scholar, and Chinese National Knowledge Infrastructure (CNKI). The source information of *Magnoliae officinalis* cortex was provided by the 2020 edition of Chinese Pharmacopoeia. The pharmacological activities, molecular mechanisms, toxicity, bioavailability, and formulations of MG are summarized, and the deficiencies of current studies are discussed.

## Pharmacological Activity

### Anti-Inflammatory Activity

Inflammation is generally characterized by overexpression of inducible nitric oxide synthase (iNOS) and cyclooxygenase-2 (COX-2) and excessive synthesis of nitric oxide (NO) and prostaglandins (PGEs) (Chen H et al., 2019). Mitogen-activated protein kinase (MAPK) and nuclear factor-κB (NF-κB) are the most crucial signaling pathways in the inflammatory process. MAPK includes four subfamilies: extracellular signal-regulated kinase (ERK), stress-activated protein kinase (SAPK)/c-Jun N-terminal kinase (JNK), big mitogen-activated protein kinase 1 (BMK1)/ERK5, and p38MAPK, which participates in cell growth, differentiation, apoptosis, immune regulation, *etc*. Furthermore, NF-κB consists of isotype or heteromorphic p50 and p65 protein, which affects the expression of inflammatory and growth factors, chemokines, COX-2, and iNOS involved in the processes of inflammation, apoptosis, tumorigenesis, *etc*. (Lu et al., 2015b). MG exhibited anti-inflammatory activity by inhibiting Toll-like receptor2 (TLR2)/TLR4/NF-κB/MAPK/peroxisome proliferator-activated receptor-γ (PPAR-γ) pathways and downregulating the expression of inflammatory cytokines (Luo et al., 2013; Wang et al., 2014; Wei et al., 2014; Lin et al., 2015; Lu et al., 2015a; Yang et al., 2016; Zhang L et al., 2018; Chen H et al., 2019; Piasecka et al., 2020).

MG (5–15 μM) could exhibit anti-inflammatory activity in lipopolysaccharide (LPS)-induced RAW 264.7 cells. It decreased the translocation of p50 and p65 subunits and downstream NF-κB transcription through downregulating inhibitor kappa B (IκB) degradation and phosphorylation. Additionally, MG blocked the phosphorylation of ERK1/2, JNK1/2, and phosphatidylinositol 3-kinase (PI3K)/protein kinase B (Akt) signal, interfered with the activation of PI3K/Akt, MAPK, and NF-κB pathway, and thus inhibited iNOS and COX-2 protein and gene expression (Lai et al., 2011). MG (5–20 μM) significantly suppressed inflammatory reaction, production of pro-inflammatory cytokines, PGE2, and nitrite, expression of iNOS and COX-2, and activation of NF-κB. Meanwhile, it elevated nuclear factor-erythroid 2-related factor 2 (Nrf2) nuclear translocation and heme oxygenase (HO)-1 expression (Lu et al., 2015b).

MG (20 mg kg^−1^, intraperitoneal (i.p.) injection) played roles in significantly ameliorating pathological characteristics and inhibiting the inflammatory reaction of acute lung injury in male Sprague Dawley (SD) rats. It could attenuate pneumonic edema, neutrophil infiltration, reactive oxygen species (ROS) production, iNOS and COX-2 expression, and NF-κB activation and upregulate PPAR-γ expression (Lin et al., 2015). MG (25 mg kg^−1^, i.p.) exhibited therapeutic effect for pathological retinal angiogenesis and glial dysfunctions by decreasing the expression of inflammatory cytokines and inactivating the HIF-1α/VEGF pathway (Yang et al., 2016).

The above results showed that MG has the effect of treating inflammation. However, most of the studies lacked positive groups. Positive groups should be set in follow-up studies.

### Antitumor Activity

In the past few decades, in order to elucidate the molecular mechanisms of tumor formation and tumorigenesis and explore therapeutic methods, a mass of studies have been done. Currently, commonly used treatment methods include radiotherapy, chemotherapy, and surgery. However, present chemotherapeutic drugs have adverse reactions such as vomiting, hair loss, kidney damage, and bone marrow destruction. It is an important challenge to find effective and economic antitumor drugs with minimum side effects. A large number of literature studies have shown that MG has antitumor activity against colon cancer (Kang et al., 2012; Park et al., 2012), prostate cancer (McKeown et al., 2014), liver cancer, lung cancer (Seo et al., 2011; Shen et al., 2017), gastric cancer (Rasul et al., 2012), cholangiocarcinoma (Zhang FH et al., 2017), oral cancer (Hsieh et al., 2018), ovarian cancer (Chuang et al., 2011), breast cancer (Liu et al., 2013), and melanoma (Cheng et al., 2020)*.* MG suppressed the growth, migration, and invasion of tumor cells and promoted apoptosis as well as autophagy by acting on caspase-8, caspase-3, and other proteins participated in the p53, MAPK, NF-κB, TLR, HIF-1α/VEGF, PI3K/Akt/ERK/mammalian target of rapamycin (mTOR), and Wnt/β-catenin signaling pathways (Chen et al., 2013; Liu et al., 2013; Li et al., 2015; Shen et al., 2017; Zhang P et al., 2017).

*In vitro*, MG (80 μM) showed the activity of suppressing the proliferation of PC3 cells (McKeown and Hurta, 2015). It could decrease the protein expression of ornithine decarboxylase, R2 subunit of ribonucleotide reductase, p-p38, JNK-1/2, PI3Kp85, p-PI3Kp85, p-Akt, NFκBp65, p-IκBα, and IκBα and increase the expression of p-JNK-1 and c-Jun. MG (10–30 μM) inhibited BRAF/mitogen-activated protein kinase (MEK) and induced cell death in melanoma via significantly downregulating PI3K/Akt pathway, which brought about a reduction of the active histone mark H3K4me3. The combination of MG and BRAF/MEK inhibitors dabrafenib/trametinib or docetaxel could have a synergistic effect (Emran et al., 2019). In MCF-7 cells, MG (20 μM) increased the expression of the tumor suppressor miRNA miR-200c to inhibit zinc finger E-box-binding homeobox 1 and increased the expression of E-cadherin (Hagiwara et al., 2015; Biersack, 2018). MG (40 μM) regulated the NF-κB pathway, induced cell cycle arrest, downregulated cyclin D1, and inhibited the expression of proliferating cell nuclear antigen (PCNA), Ki67, matrix metalloproteinase (MMP)-2, MMP-7, and MMP-9 to control the growth, migration, and invasion of QBC939 cells (Zhang FH et al., 2017). In A549 cells, MG (1–50 μM) showed growth inhibition and autophagy via activating caspase-3 and poly-(ADP)-ribose polymerase cleavage, reducing NF-κB/Rel A and Akt/mTOR pathway expression, dose-dependently blocking mitosis and G2/M progression, and increasing the release of lactate dehydrogenase (Liu et al., 2013; Shen et al., 2017). What is more, in OC2 cells, MG (20–100 μM) played roles of [Ca2+] increase, phospholipase C-dependent Ca2+ release from the endoplasmic reticulum, Ca2+ entry, and Ca2+-independent cell death (Hsieh et al., 2018). In U87MG and LN229 human glioma cells, cotreatment with MG and honokiol exerted a synergistic antitumor effect to induce cell cycle arrest as well as autophagy and inhibit proliferation by decreasing cyclin A/D1, cyclin-dependent kinase 2, 4, 6, p-PI3K, p-Akt, Ki67, p-p38, and p-JNK and elevating p-ERK expression (Cheng et al., 2016).

*In vivo*, MG (5–20 mg kg^−1^, i.p. injection) inhibited the growth of GBC-SD tumor in BALB/c nude xenograft model. It significantly increased caspase-3 activation and inhibited cell division cycle gene (CDC) 2 expression (Li et al., 2015). In addition, treated with MG (40 mg kg^−1^, i.p. injection) in the nude immune-deficient mice, it could be observed that the growth of nude immune-deficient MDA-MB-231 and MCF-7 tumors was inhibited, and the level of MMP-9 was decreased (Liu et al., 2013). In the human GBM orthotopic xenograft model, compared with temozolomide, cotreatment with MG and honokiol could more effectively inhibit tumor progression and induce apoptosis (Cheng et al., 2016).

In a word, MG and honokiol suppress the proliferation, migration, and invasion of tumor cells and promote apoptosis as well as autophagy by regulating MAPK, NF-κB, HIF-α, PI3K/Akt/ERK/mTOR, and Wnt/β-catenin signaling pathways (Tse et al., 2005; Vavilala et al., 2014; Lin et al., 2016; Lee et al., 2019). In addition, MG shows antitumor activity by regulating TLR signaling pathways. Honokiol also can regulate STAF, EGFR, and notch signaling pathways to exhibit antitumor activities (Leeman-Neill et al., 2010; Liu et al., 2012; Kaushik et al., 2015). Further experiments *in vivo* are needed, and attention should be paid to whether MG could cause side effects.

### Antiangiogenesis Activity

Angiogenesis, the essential procedure of embryonic angiogenesis, organ regeneration, and wound healing, is involved in many pathological illnesses, such as cancer, rheumatoid arthritis, and diabetic retinopathy. It is of great significance to study the molecular mechanism of angiogenesis, find relevant new drugs, and provide potential lead candidates. Studies have shown that ROS can participate in the signal transduction cascade in the key steps of angiogenesis and regulate the growth and migration of endothelial cells. MG inhibited angiogenesis through regulating the PI3K/Akt/mTOR signaling pathway and HIF-1α/vascular endothelial growth factor (VEGF)-dependent pathway and inhibiting ROS production (Kim GD et al., 2013; Chen et al., 2013).

MG (10 μM) reduced the accumulation of HIF-1α protein by enhancing the activity of prolyl hydroxylase and reducing the synthesis of HIF-1α protein (Chen et al., 2013). MG (20 μΜ) has been shown to significantly inhibit the transcription and translation activity of platelet endothelial cell adhesion molecules and induce the production of ROS by mediating mitochondria and apoptosis. Furthermore, MG inhibited the activation of MAPKs and PI3K/Akt/mTOR signaling pathways in mouse embryonic stem (MES)/embryoid body (EB)-derived endothelial-like cells (Kim GD et al., 2013). MG (10 and 40 μM) suppressed the proliferation of human umbilical vein endothelial cells (HUVECs), ERK1/2 activity, gelatinase activity, and production of ROS and promoted HO-1 levels (Kuk et al., 2017).

In the T24 xenograft mouse (C57BL/6 mice), MG (5–10 mg kg^−1^, i.p. injection) inhibited angiogenesis, tumor proliferation, and the expression of HIF-1α, VEGF, endothelial cell marker CD31, and endogenous hypoxia biomarker carbonic anhydrase IX by suppressing HIF-1α/VEGF-dependent pathway (Chen et al., 2013). MG (20 μg/ear) was transdermally administered to male NMRI mice. It inhibited the venous remodeling process and decreased endothelial proliferation and MMP-2 abundance by amplifying the HO-1-mediated resistance of endothelial cells to ROS-mediated proliferative stimuli and blocking the proteolytic activity upon biomechanical load (Kuk et al., 2017).

### Cardiovascular Protection

Cardiovascular disease is a large class of diseases, including coronary artery disease, hypertension, dyslipidemia, congenital heart disease, valve disease, and arrhythmia. With the improvement of people's living standards, the incidence of cardiovascular diseases is gradually increasing. MG showed activities of inhibiting the migration and hyperplasia of vascular smooth muscle cells (VSMCs), such as antiplatelet, antithrombotic, and antihypertensive via inhibiting MAPK family activation, Akt/ERK1/2/GSK3 β-catenin pathway, and angiotensin-converting enzyme (ACE)/angiotensin II (Ang II)/Ang II type 1 receptor (AT-1R) cascade and upregulating PPAR-β/γ and NO/guanosine 3′,5′-cyclic phosphate/PKG pathways (Shih and Chou, 2012; Karki et al., 2013b; Liang X et al., 2014; Wu et al., 2015; Chang et al., 2018).

Under pathological conditions, the proliferation and migration of VSMCs to the intima can lead to vascular diseases such as atherosclerosis and restenosis after balloon angioplasty (Karki et al., 2012). MG (20 and 30 μM) inhibited VSMCs migration, β1-integrin expression, focal adhesion kinase (FAK) phosphorylation, RhoA and cell division cycle 42 (Cdc42) activation, and collagen-induced stress fiber formation (Karki et al., 2013b). MG (20 μM) suppressed VSMC proliferation and DNA synthesis by inhibiting the expression of cyclin D1/E and cyclin-dependent kinase 2 and 4, ROS production, and activation of renin–angiotensin system, MEK, and ERK1/2 (Karki et al., 2013a; Wu et al., 2015). Additionally, it (1–100 μM) could play the role of vasodilator and eliminate superoxide anion by relaxing right coronary arteries (separated from hearts of pigs) in a dose-dependent manner and controlling the expression levels of iNOS and COX-2, with an IC_50_ value of 5.78 μM (Kuo et al., 2011). Further pharmacological research in this field was needed to reveal the mechanism by which MG inhibited homocysteine-induced endothelium-dependent vasodilation damage.

*In vivo*, MG (50 and 100 mg kg^−1^, gavage) caused attenuation of neointima formation, intimal area, and intimal/medial ratio and increase of luminal area via significantly decreasing the expression of cyclin D1/E and CDK4/2 mRNA and protein (Karki et al., 2013a). In male SD rats with pulmonary hypertension (PHA), MG (100 mg kg^−1^, i.p. injection) exerted a therapeutic effect of PHA by altering the Akt/ERK1/2/glycogen synthase kinase 3β (GSK3β)-catenin pathway. It upregulated ACE2 and significantly downregulated the expression of iNOS, endothelin-1 (ET-1), and ETA receptors and O^2-^ production (Chang et al., 2018).

### Hypoglycemic Activity

Diabetes is a metabolic disease characterized by hyperglycemia, which is caused by insufficient insulin excretion and impaired biological effects. Long-term hyperglycemia can contribute to chronic injury and dysfunction in numerous tissues, especially eyes, kidneys, and heart. Type 2 diabetes, formerly known as adult-onset diabetes, mostly occurs after 35–40 years of age and accounts for more than 90% of diabetic patients (Maddaloni et al., 2020). Numerous studies have reported that MG exhibits the hypoglycemic activity and protein tyrosine phosphatase 1B (PTP1B) inhibition by mediating AMPK/silent information regulator 1 (SIRT1)/PGC-1α, PPAR-γ, and protein kinase A (PKA) pathways, enhancing the activities of glyoxalase 1, PDX1, Ins2, and GPX genes, stimulating Akt phosphorylation, and inhibiting α-glucosidase (Choi et al., 2012; Wang HY et al., 2013; Onoda et al., 2016; Pulvirenti et al., 2017; Suh et al., 2017; Parray et al., 2018).

Low-dose MG (0.01–1 μM) inhibited the death of RIN-m5F cells and the decrease of insulin secretion induced by methylglyoxal, thereby exerting hypoglycemic activity (Suh et al., 2017). It could upregulate the expression of Ins2 and PDX1, the levels of SIRT1 and PGC1α, AMPK phosphorylation, and glyoxalase 1 activity. Moreover, it attenuated the level of methylglyoxal-modified protein adducts and protected protein glycosylation (Alonso-Castro et al., 2011). In L6 myotubes, honokiol (3–30 μM) and MG (3–30 μM) stimulated glucose uptake in a dose-dependent manner and promoted the translocation of glucose transporter-4 to the cell surface as well as Akt phosphorylation. Their activity to stimulate glucose uptake could be blocked by the phosphatidylinositol 3-kinase inhibitor, wortmannin (Choi et al., 2012). MG (20 μM) reduced metabolic disorders, oxidative stress, and fat formation by promoting the adipocyte differentiation and browning of 3T3-L1 C3H10T1/2 cells adipocyte-specific marker genes (uncoupling protein 1, CD137, Tbx1, *etc.*) and protein expression (Parray et al., 2018). It upregulated key fatty acid oxidation and lipid biomarkers (carnitine palmitoyltransferase 1C, acyl-CoA synthase long-chain family member 1, SIRT1, and perilipin) and activated AMPK, PPAR-γ, and PKA pathways. Honokiol and MG inhibited α-glucosidase with IC_50_ values of 2.3 and 0.4 µM, respectively (Wang HY et al., 2013). Moreover, their inhibition at 1.5 μM was 3.9 and 29.8%, respectively (Pulvirenti et al., 2017). The inhibitory effect of honokiol on α-glucosidase was lower than that of MG.

C57BL/6J mice were fed a high-fat diet (45 kcal% fat) with or without honokiol (0.02%, w/w) or MG (0.02%, w/w) for 16 weeks. The results showed that honokiol and MG significantly lowered the weight of white adipose tissue, adipocyte size, and proinflammatory gene expression, protected against insulin resistance, and elevated plasma IL-10 level. In particular, honokiol could significantly decrease the plasma resistin level and increase the plasma adiponectin level compared to the control group (Kim YJ et al., 2013).

It can be seen that MG and honokiol have similar mechanisms to play a hypoglycemic role, such as inhibition of α-glucosidase and stimulation of glucose uptake. The difference is that MG has a better inhibitory effect on α-glucosidase, while honokiol can significantly decrease the plasma resistin level and increase the plasma adiponectin level.

### Gastrointestinal Protection

*In vitro*, MG (3–100 μM) inhibited the spontaneous contraction, acetylcholine (ACh)- and Bay k8664-induced contraction, L-type Ca^2+^ current, and the contraction of colonic smooth muscle through decreasing L-type Ca^2+^ channel activity (Zhang et al., 2013).

In the Kunming mouse model of diarrhea induced by castor oil, MG (25, 50, and 100 mg kg^−1^, gavage) significantly inhibited diarrhea, reduced small intestinal transport, and increased catalase (CAT), superoxide dismutase (SOD), and glutathione peroxidase (GSH-Px) (Pang et al., 2013). Zeng *et al.* found that the antidiarrheal mechanism of MG and honokiol was similar, but *in vivo* experiments showed that MG had a higher antidiarrheal activity than honokiol (Zeng et al., 2015). The reason might be related to the inhibition of the liver CYP450 enzyme. Deng *et al.* reported that MG (100, 300, and 500 mg kg^−1^, gavage) and honokiol (100, 300, and 500 mg kg^−1^, gavage) regulated the release of IP3-Ca2+ storage, suppressed SK channel, and facilitated the opening of BKα1 as well as BKβ3 channels and the closing of BKβ4 channel by blocking the IP3-Ca2+ channel, inhibiting the activation of IP3 receptor 1 and CaM, and regulating protein kinase C (PKC) (Deng et al., 2015). In this study, the dose of MG and honokiol was too high and there was no positive control, so the dose should be reduced, and a positive control should be set for further research.

In conclusion, both MG and honokiol can exhibit gastrointestinal protective activity with similar mechanism, while MG’s antidiarrheal activity is better than that of honokiol.

### Neuroprotection

It is worth noting that MG can cross the blood–brain barrier (BBB) (Ranaware et al., 2018). A great quantity of research studies has demonstrated that it has generous pharmacological activities in the nervous system. Cannabinoid (CB) receptors are composed of CB1 and CB2 (Geiger et al., 2010). CB1 receptor activation is involved in the regulation of memory, cognition, and motor control, for example, relieving pain, vomiting, reducing hyperexcitability in epilepsy, stimulating appetite, and euphoria. CB2 receptor activation brings about antinociceptive and inflammatory activities (Fuchs et al., 2013). Studies have found that MG was a partial agonist of CB1 (EC_50_ = 18.3 ± 8.6 µM) and CB2 (EC_50_ = 3.28 ± 2.10 µM), while honokiol was a full agonist of CB1 (EC_50_ > 10 µM) and an inverse agonist of CB2. 4′-O-Methylhonokiol was a CB2 receptor agonist and a potent COX-2 SSI (Chicca et al., 2015). In addition, MG had no activity on GPR-55, while honokiol was an antagonist of GPR-55 (Rempel et al., 2013; Coppola and Mondola, 2014; Fuchs et al., 2014). MG showed a certain preference for CB2 in binding studies with Ki values for CB1 and CB2 of 3.19 and 1.44 µM, respectively. Ki values of honokiol at CB1 and CB2 were 6.46 and 5.61 µM, respectively (Schuehly et al., 2011; Rempel et al., 2013). The Ki values of 4′-O-methylhonokiol at CB1 and CB2 were 2.4 μM and 188.5 nM, respectively (Chicca et al., 2015). MG played an antidepressant role by adjusting the hypothalamic–pituitary–adrenal (HPA) axis and hippocampal neurotransmitters and increasing the expression levels of brain-derived neurotrophic factor (BDNF), serotonergic system activity, such as nerve inflammation, and the prefrontal cortex oxidative stress (Li et al., 2012, Li LF et al., 2013; Matsui et al., 2016; Bai et al., 2018; Cheng et al., 2018). MG was a dual agonist of PPAR-γ (EC_50_ = 0.93 ± 0.91 µM) and RXRα (EC_50_ = 3.91 ± 1.08 µM) (Dreier et al., 2017). In addition, MG and honokiol could improve both phasic and tonic GABAergic neurotransmission in hippocampal dentate granule neurons (Alexeev et al., 2012). Honokiol had a stronger positive regulatory effect on GABAA receptors than MG (Fuchs et al., 2014). In α1β2γ2 receptor and β1 containing subtype, the EC_50_ value was at approximately 20 μM for honokiol (Rycek et al., 2015). MG exhibited the activities of anti-AD, antiepileptic, and neuroprotection by acting on PPAR-γ targets, GABAA/benzodiazepine receptor complex, NF-κB, JNK/mitochondrial/caspase, and PI3K/MEK/ERK/Akt/forkhead transcription factor (FoxO) 1 pathways, alleviating inflammation, promoting microglia phagocytosis and Aβ degradation, reducing the seizure mortality, prolonging seizure time, and inhibiting apoptosis (Chen CR et al., 2011; Wang CC et al., 2013; Chen et al., 2014; Chen et al., 2014; Rycek et al., 2015; Kou et al., 2017; Kou et al., 2017; Zhou F et al., 2019; Zhou F et al., 2019; Li J et al., 2020; Xie et al., 2020).

In BV2 cells, MG (10 μM) attenuated Aβ-induced AD by inhibiting the luciferase activity of NF-κB and the target gene of inflammatory cytokines, activating luciferase and liver X receptor activity, reducing ROS production induced by Aβ, upregulating apolipoprotein E (ApoE), and promoting microglial phagocytosis and Aβ degradation (Xie et al., 2020). MG (EC_50_ = 3.49 μM) and honokiol (EC_50_ = 2.65 μM) promoted the transcriptional activities of PPAR-γ in a dose-dependent manner. They also dose-dependently increased the luciferase activity of PPAR-γ-LBD. MG and honokiol could fit into the protein pocket of PPAR-γ-LBD with IC_50_ values of 3.745 and 16.13 μM, respectively. What is more, MG had two hydrogen bonds at Glu343, which maintained the binding stability, while honokiol had one hydrogen bond at Glu343 and SER342, respectively, indicating that MG was more effective in enhancing PPAR-γ luciferase levels than honokiol (Xie et al., 2020). MG (5 μM) significantly inhibited trimethyltin (TMT)-mediated neuronal death and microglial activation by inhibiting ROS production and the activation of JNK, p38 MAPKs, and NF-κB in HT22 cells and BV-2 cells (Kim and Kim, 2016). Both MG (12.5 μM) and honokiol (6.25 μM) showed effective behavioral and electrophysiological antiepileptic activities in pentylenetetrazole and ethyl ketopentenoate models (Li G et al., 2020).

At concentrations of 50 and 100 mg kg^−1^, MG alleviated depression-like behavior in male ICR mice by reducing corticosterone (CORT) level and increasing NE, 5-hydroxytryptamine (5-HT), and BDNF protein levels (Bai et al., 2018). It could improve depressive behavior and hippocampal nerve damage in male ddY mice (Matsui et al., 2016). The phosphorylation of Akt, ERK, and cyclic AMP-responsive element-binding protein was significantly increased. In a male Kunming mouse model of chronic mild stress (CMS), MG (20 and 40 mg kg^−1^, gavage) downregulated the levels of interleukin-1β (IL-1β), IL-6, and tumor necrosis factor-α (TNF-α) in the prefrontal cortex, suppressed the activation of microglia and the proliferation of HPA axis and oxidative stress, and reversed malondialdehyde increase and SOD as well as GSHPx decrease to produce antidepressant-like effect (Cheng et al., 2018). MG (10 and 30 mg kg^−1^, i.p. injection) downregulated the expression of bax and Ac-FOXO1 and production of NOS, 4-HNE, iNOS, phosphorylated p38MAPK, and C/EBP homologs, while upregulated the expressions of Bcl-2 and SIRT1. The regulation effect of MG on ischemic damage factors may be through inhibiting the production of ROS and upregulating p-Akt and NF-κB (Chen et al., 2014). MG (40 and 80 mg kg^−1^) exhibited antiepileptic activity by prolonging the latency of seizure onset and decreasing the number of seizure spikes, through acting on GABAA/benzodiazepine receptor (Chen CR et al., 2011).

As indicated by the above results, both MG and honokiol can act on CB1 and CB2 receptors. The difference is that MG is a partial agonist of CB1 and CB2, while honokiol is a full agonist of CB1 and an inverse agonist of CB2, and MG has no activity on GPR-55, while honokiol is an antagonist of GPR-55. MG and honokiol can improve both phasic and tonic GABAergic neurotransmission in hippocampal dentate granule neurons; however, honokiol has a stronger positive regulatory effect on GABAA receptors than MG. In addition, MG and honokiol promote the transcriptional activities of PPAR-γ in a dose-dependent manner. They also dose-dependently increased the luciferase activity of PPAR-γ-LBD. However, MG is more effective in enhancing PPAR-γ luciferase levels than honokiol. MG had antidepressant, anti-AD, anticonvulsant, anti-neurological deterioration, and protective effects to brain injury in the nervous system. Honokiol can regulate CB2 receptor, PPAR-γ targets, GABAA, and NF-κB and inhibit the levels of IL-Iβ, IL-6, IL-8, and TNF-α, production of ROS, RNS, COX2 as well as iNOS, and expression of PI3K/Akt, MAPKs, ERKs, JNKs, and p38 to exert neuroprotective effects (Talarek et al., 2017).

### Interaction with CYP450 Enzyme

CYP450 is an important enzyme system involved in drug metabolism *in vivo* (Totah and Rettie, 2005). Among them, CYP2C8, CYP2C9, CYP2E1, and CYP2A6 accounted for about 40% of the total CYP450 enzymes in the liver (Zhang P et al., 2017). It is of great significance to study the interaction between the active components of traditional Chinese medicine and CYP450 for clinical safety. Studies have shown that MG can inhibit many CYP enzymes in humans and rats.

The IC_50_ values of MG on human CYP1A, CYP2C, CYP3A, CYP3A4, CYP2C19, CYP2C8, and CYB2C6 were 5.56, 41.48 ± 5.13, 35.0, 56.2, 0.527, 1.62, and 44.9 μM, respectively. And the IC_50_ values of MG on rat CYP2C, CYP2D6, CYP2E1, CYP3A4, CYP1A2, CYP2B6, CYP1A, CYP3A, CYP2C11, and CYP2D were 5.56 ± 2.87, 65.42 ± 4.46, 67.93 ± 9.51, 52.36 ± 17.32, 97.80 ± 3.83, 28.69 ± 1.46, 5.56, 3.8, 84.5, and 39.9 μM, respectively. In addition, IC_50_ values of CYP2C8, CYP2A1, and CYP2A2 in rat liver and CYP2E1 and CYP2A6 in the human liver were greater than 100 μM. The inhibition types of MG on CYP1A (Ki: 1.09–12.0 μM), CYP2C19 (Ki: 0.449 μM), CYP2C (Ki: 10.0–15.2 μM), 3A (Ki: 93.7–183 μM), and CYP1A2 (Ki: 10.0 μM) were competitive inhibition. The IC_50_ values of honokiol on human CYP1A2, CYP2B6, CYP2C8, CYP2C9, CYP2C19, CYP2D6, CYP3A, and CYP3A4 were 3.5, 18.8, 40.8, 9.6, 32.9, >50, >50, and 43.9 μM, respectively. Moreover, the IC_50_ values of honokiol on rat CYP2C, CYP2A6, CYP2D6, CYP2E1, CYP3A4, CYP1A2, and CYP2B6 were 41.86 ± 4.24, >100, 43.43 ± 2.34, 58.10 ± 3.02, >100, 95.24 ± 7.81, and 53.22 ± 0.66 μM, respectively. The inhibition type of honokiol on CYP1A2 (Ki: 6.2 μM) was competitive inhibition, and the inhibition types of honokiol on CYP2E1 (Ki: 11.1 μM) and CYP2C19 (Ki: 0.702 μM) were noncompetitive inhibition (Joo and Liu, 2013; Duan et al., 2015; Kim SY et al., 2015; Liu et al., 2016; Zhang P et al., 2017; Huang et al., 2019; Kim S. B. et al., 2015)

Kim *et al.* proved the feasibility of MG and honokiol to modulate CYP activity *in vivo* by using the phenacetin and diclofenac as probe substrates for rat CYP1A and 2C, respectively. The result indicated that the mean IC_50_ values of MG for the metabolism of phenacetin and diclofenac were 19.0 and 47.3 µM, while those of honokiol were 8.59 and 44.7 µM, respectively. The inhibitory effect of MG and honokiol on CYP1A activity was stronger than that of CYP2C activity rat liver microsomes (Kim SB et al., 2018). Huang *et al.* revealed that different CYP450 enzyme isoforms showed different activities in the *in vitro* metabolism of MG and honokiol in rat liver microsomes (Huang et al., 2019). The CYP2E1 subtype managed the oxidation of MG and honokiol terminal double bonds to epoxy metabolites, CYP3A4 seemed to be the main subtype responsible for further hydrolytic metabolism, while CYP1A2 might promote the decarboxylation of metabolites. CYP2A6 might be the key subtype leading to MG hydrogenation. It is necessary to further study the pharmacokinetic interaction between MG and CYP substrate drugs *in vitro* and *in vivo*.

### Antibacterial Activity

According to the literature review, MG has antibacterial activities. It could inhibit the *Aeromonas hydrophila* strains, with the minimal inhibitory concentration (MIC) value range of 32–64 μg ml^−1^ (Dong et al., 2017). MG and honokiol exhibited similar inhibitory activity against methicillin-resistant *Staphylococcus aureus* (MRSA) and methicillin-susceptible *S. aureus* (MSSA), with the MIC/minimal bactericidal concentration (MBC) value range of 16–64 mg L^−1^ (Zuo et al., 2015). Honokiol and MG dose-dependently inhibited the MRSA strain with the MIC values of 33 and 20 μg ml^−1^, respectively (Kim SY et al., 2015). They inhibited multidrug-resistant and MRSA with MIC values in the range of 8–16 ppm (Liu et al., 2014). Choi *et al.* reported that honokiol and MG caused significant cellular immune-modulatory effect and decreased the production of ROS and inflammatory cytokines/chemokines during *S. aureus* infection. Honokiol upregulated type I and III interferon mRNA expression in response to MSSA infection and inhibited the growth of MSSA at 2.5 μg ml^−1^ and MRSA at 5 μg ml^−1^, whereas MG inhibited the growth of both bacterial cells at 5 μg ml^−1^ after 24 h of growing (Choi et al., 2015). MG and honokiol could inhibit *S. mutans* to prevent dental caries, with an MIC value of 10 μg ml^−1^. And MG (50 μg ml^−1^) had better bactericidal activity against *S. mutans* biofilm than honokiol (50 μg ml^−1^) and chlorhexidine (500 μg ml^−1^) at 5 min after exposure (Sakaue et al., 2016).

In addition, in the seven pathogenic fungi including *Alternaria alternata* (Fr.) Keissl, *Penicillium expansum* (Link) Thom, and *Alternaria dauci* F.Sp. solani, MG inhibited their growth by more than 57% (Chen Y-H et al., 2019). Moreover, the MIC value ranged from 16 to 64 g ml^−1^ for the 64 *Candida* spp. strains, and the MICs of *Candida* CSC*27907, CDC27897, CDC28621, and ATCC24433 were 64, 32, 16, and 32 μg ml^−1^, respectively. And the average inhibition rate of biofilm was 69.5% (Behbehani et al., 2017). Honokiol exhibited better antimicrobial activity than MG on *Aggregatibacter actinomycetemcomitans*, *S. mutans*, *S. aureus*, MRSA, and *Escherichia coli* with MIC/MBC values of 10/10, 10/20, 10/20, 10/90, and > 100/> 100 g ml^−1^, respectively, while those of MG were 10/20, 10/20, 10/30, 20/90, and > 100/> 100 g ml^−1^, respectively (Chiu et al., 2020).

Oufensou *et al*. tested the antifungal activities of MG and honokiol (5–400 μg ml^−1^) against 32 *Fusarium* spp. strains. The terbinafine (0.1–10 μg ml^−1^) and fluconazole (1–50 μg ml^−1^) were used as positive controls. The results revealed that MG had similar bactericidal activity compared with fluconazole, whereas honokiol had a better effect of inhibiting the mycelium growth compared to this fungicide. Compared to terbinafine, honokiol exhibited similar antifungal activity, whereas MG was less effective at all selected concentrations (Oufensou et al., 2019).

### Antioxidant Activity

Amorati *et al.* explored the chemistry behind the antioxidant activity of MG and honokiol. They found that MG trapped four peroxyl radicals, with a *k*inh of 6.1 × 104 M^−1^ s^−1^ in chlorobenzene and 6.0 × 103 M^−1^ s^−1^ in acetonitrile, while honokiol trapped two peroxyl radicals in chlorobenzene (*k*inh = 3.8 × 104 M^−1^ s^−1^) and four peroxyl radicals in acetonitrile (*k*inh = 9.5 × 103 M^−1^ s^−1^). Their different behavior was due to the combination of intramolecular hydrogen bonding among the reactive OH groups (in MG) and of the OH groups with the aromatic and allyl π-systems (Amorati et al., 2015). MG has a bisphenol core with two allylic side chains, and its antioxidant activity is attributed to hydroxyl and allyl groups (Baschieri et al., 2017). MG downregulated myeloperoxidase (MPO) activity and the expression of TNF-α, iNOS, and IL-6 by altering JNK/mitochondrial/caspase and PI3K/MEK/ERK/Akt/FoxO1 signaling pathways (Shih et al., 2012; Dong et al., 2013).

*In vitro*, MG (16 μM) protected against acrolein-induced oxidative stress in human SH-SY5Ycells via acting on JNK/mitochondrial/caspase and PI3K/MEK/ERK/Akt/FoxO1 signaling pathways and inhibiting intracellular glutathione consumption as well as ROS accumulation (Dong et al., 2013).

It was found that MG (20 mg g^−1^, i.v. injection) could significantly reduce MPO activity and the expression of iNOS, TNF-α, and IL-6 to inhibit oxidative stress and reduce mesenteric reperfusion caused lung injury in male C3H/HeOuJ mice (Shih et al., 2012). In aristolochic acid (AA)-induced HK-2 cells, MG (10 μM) and honokiol (10 μM) effectively reduced oxidative stress and suppressed cell proliferation by blocking the cell cycle at the G1 phase and preventing the G2/M arrest (Bunel et al., 2016).

### Other Activities

Besides these pharmacological activities mentioned above, MG also has the following activities: inhibition of osteoclast differentiation, antiphotoaging, antiparasitic, antiviral activity, and reduction of multidrug resistance.

MG (0.1 μM) significantly downregulated the production of osteoclast differentiation-inducing factors such as RANKL, TNF-α, and IL-6 and inhibited mitochondrial electron transport (Kwak et al., 2012). In RANKL-induced RAW 264.7 macrophages, MG (75–150 μM) decreased osteoclast differentiation, tartrate-resistant acid phosphatase activity of differentiated cells, and resorption pit area caused by osteoclasts in a concentration-manner (Lu et al., 2013). MG (10 μM) inhibited IL-1-induced RANKL expression and osteoclast differentiation by suppressing COX-2 expression and PGE2 production (Hwang et al., 2018). MG (2.5–20 μM) attenuated RANKL-induced osteoclast differentiation by suppressing MAPK/c-fos/AP-1 and NF-κB signaling, inhibiting ROS production, and increasing HO-1 expression (Lu et al., 2015a). In tendon-derived stem cells (TDSCs), MG (5–20 μM) prevented calcium deposition and osteogenic differentiation of tendon-derived stem cells through influencing PI3K/Akt/β-catenin pathway induced by PEG-2 (Zhou W et al., 2019). In ligature-induced rats, MG (100 mg kg^−1^, p.o.) significantly suppressed alveolar bone resorption, the number of osteoclasts on the bony surface, and the expression of RANKL. Moreover, it could reduce the expression of MMP-1, MMP-9, iNOS, and COX-2 and TNF-α activation (Lu et al., 2013). MG (25 mg kg^−1^, i.p. injection) inhibited the activities of osteogenic factors runt-related transcription factor 2(RUNX2), OCN, and bone morphogenetic protein 2 (BMP2) in male SD rats. Moreover, it inhibited ossification of tendon ossification by reducing heterotopic ossification of Achilles tendon (Zhou W et al., 2019).

After treating HR-1 hairless male mice with 40 μL of the 0.25% MG preparation, it significantly reduced the average length and depth of wrinkles and inhibited the expression of MMP-1, MMP-9, and MMP-13 to play a role in antiphotoaging activity (Im et al., 2015).

MG significantly inhibited HBV activities. The IC_50_ values of HBV surface antigen (HBsAg), HBV e antigen (HBeAg), and replication of HBV DNA were 2.03, 3.76, and 8.67 μM, respectively, and without cytotoxicity to HBsAg and HBeAg (Li J et al., 2013). MG (2.51 ± 0.51 μg ml^−1^) and honokiol (3.18 ± 0.61 μg ml^−1^) stimulated the expression of immune-related genes to resist grass carp reovirus infection in *Ctenopharyngodon idella* kidney (CIK) cells. MG significantly increased the expression of interferon (IFN) regulatory factor (IRF) 7 and IL-1β to activate type I IFN (IFN-I) but failed to induce the molecules in NF-κB pathways. The difference was that honokiol promoted the expression of IL-1β, TNFα, NF-κB, IFN-β, promoter stimulator 1, IRF3, and IRF7 but failed to increase IFN-I expression, showing that it could enhance the host innate antiviral response to grass carp reovirus infection by regulating NF-κB pathway (Chen et al., 2017).

What is more, MG (1–50 μM) reduced the multidrug resistance of cancer cells to antitumor drugs through downregulating P-glycoprotein expression in a concentration- and time-dependent manner and increased the intracellular accumulation of calcein in NCI/ADR-RES cells (Han and Van Anh, 2012).

## Toxicity

So far, a large number of studies have shown that MG has cytotoxicity (Table 2). MG (10–100 μM, 24 or 48 h) was used to investigate the toxicity on human normal hepatocyte U937 and LO-2 cells. The results showed that MG at low concentration could promote the cell survival rate in a dose-dependent manner. At a concentration of less than 60 μM, MG could promote the survival of U937 cells. When exposed to MG at a concentration of less than 70 μM after 48 h, the mortality of LO-2 cells was lower than 20% (Chen H et al., 2019). Additionally, at a concentration range from 50 to 200 μg ml^−1^, MG could cause toxicity and inhibit MMEC survival (Wei et al., 2014).

**TABLE 2:** Toxicity of MG.

Activity	Cell lines	Dosage	Application	References
Inhibition of cell viability	U937 and LO-2 cells	10–100 μM	*In vitro*	Chen H et al. (2019)
Inhibition of cell viability	MMECs	50–200 μg ml^−1^	*In vitro*	Wei et al. (2014)
Inhibition of cell migration	VSMCs	40 μM	*In vitro*	Karki et al. (2013a)
Inhibition of cell viability	Murine 3T3-F442A predipocytes and human normal subcutaneous predipocytes	30–100 μM	*In vitro*	Alonso-Castro et al. (2011)
Inhibition of cell growth	mES-derived endothelial-like cells	50–100 μM	*In vitro*	Kim GD et al. (2013)
Inhibition of cell viability	MCF-10A, MCF-7, SK-BR3, MDA-MB-453, MDA-MB-435S, MDA-MB-231, and MDA-MB-468 cells	IC_50_: 70.52 ± 5.09, 36.46 ± 2.38, 59.40 ± 8.24, 35.69 ± 4.91, 25.39 ± 3.26, 25.32 ± 2.72, and 24.79 ± 3.06 μM, respectively	*In vitro*	Liu et al. (2013)
Inhibition of cell viability	HCT-116 cells	1–50 μM	*In vitro*	Park et al. (2012)
Inhibition of cell viability	OC2 cells	20–100 μM	*In vitro*	Hsieh et al. (2018)
Inhibition of cell viability	A549 cells	6.25–200 μM	*In vitro*	Wu et al. (2014)
Inhibition of cell viability	DU145 and PC3 cells	40 and 80 μM	*In vitro*	McKeown et al. (2014)
Induction of cell apoptosis	GBC cells	10–30 μM	*In vitro*	Li et al. (2015)
Induction of cell apoptosis	SGC-7901 cells	10–300 μM	*In vitro*	Rasul et al. (2012)
Inhibition of cell proliferation	SKOV3 and TOV21G cells	6.25–100 μM	*In vitro*	Chuang et al. (2011)
Inhibition of cell proliferation	QBC939, SK-ChA-1, MZ-ChA-1, and RBE cells	20–160 μM	*In vitro*	Zhang FH et al. (2017)

Karki *et al.* reported that MG at a concentration of 40 μM possessed cytotoxicity on VSMCs (Karki et al., 2013a). MG (100 μM) reduced the murine 3T3-F442A preadipocyte viability by 25% and human normal subcutaneous preadipocyte viability by 36%. MG (50 μM) reduced the murine cell viability by 16% and human cell viability by 22%. Otherwise, honokiol (50 μM) significantly decreased the murine and human cell viability by 30 and 39%, and the combined application of honokiol and MG (100 μM each) markedly decreased the cell viability by 73% (murine) and 80% (human). The combined application of honokiol and MG (50 μM each) also markedly reduced murine (31%) and human (37%) cell viability. On the contrary, the simultaneous application of honokiol and MG (30 μM each) only moderately affected the murine (15%) and human (21%) cell viability (Alonso-Castro et al., 2011). When the concentration of MG was > 50 μM, it would be toxic to mES-derived endothelial-like cells (Kim GD et al., 2013). Liu *et al.* studied the cytotoxicity of MG on human breast cancer cell lines and normal human mammary epithelial cells. The results showed that MG had moderate cytotoxicity to MCF-10A, MCF-7, SK-BR3, MDA-MB-453, MDA-MB-435S, MDA-MB-231, and MDA-MB-468 cells with IC_50_ values of 70.52 ± 5.09, 36.46 ± 2.38, 59.40 ± 8.24, 35.69 ± 4.91, 25.39 ± 3.26, 25.32 ± 2.72, and 24.79 ± 3.06 μM, respectively (Liu et al., 2013). Park *et al.* treated HCT-116 colon cancer cells with various concentrations of MG (0–50 µM) for 24 and 48 h. MG induced cell death in a dose- and time-dependent manner. Treatment with 50 μM MG for 24 h resulted in significant decreases in cell viability with 75.3% of the cells surviving after 24 h and 81.7% of the cells surviving after 48 h. Moreover, MG (50 µM) induced apoptosis in 76.1% of the cells after 24 h, indicating that MG inhibited cell proliferation and induced apoptosis in HCT-116 cells (Park et al., 2012). When OC2 cells were treated with MG (20–100 μM) for 24 h, the cell viability decreased in a dose-dependent manner (Hsieh et al., 2018). After treating A549 cells with 6.25, 12.5, 25, 50, 100, and 200 μM of MG for 24 and 48 h, cell viability for 24 h was 98.1 ± 2.7, 86.4 ± 2.3, 79.5 ± 4.6, 68.7 ± 2.3, 55.9 ± 1.1, and 12.8 ± 3.1%, respectively, while for 48 h was 92.5 ± 3.5, 80.1 ± 4.7, 70.2 ± 2.8, 56.6 ± 3.4, 36.3 ± 2.6, and 3.1 ± 0.9%, respectively. When the dose of MG was ≤6.25 μM, there was almost no inhibitory effect on A549 cells, while 25 μM of MG significantly inhibited the proliferation of A549 cells. MG inhibited the proliferation of A549 cells in a dose- and time-dependent manner (Wu et al., 2014). In DU145 cells, the viability was reduced by 30 and 60% at 40 and 80 μM, respectively, after 6 h of MG treatment, and 49 and 76% were reduced at 40 and 80 μM, respectively, after 24 h of MG treatment. After treating PC3 cells with 80 μM MG for 6 and 24 h, its viability decreased to 50 and 48%, respectively (McKeown et al., 2014). Li et al. treated GBC cells with MG at concentrations of 10, 20, and 30 μM for 48 h. The results showed that the apoptosis index of GBC cells was significantly higher than that of the control group (Li et al., 2015). SGC-7901 cells were treated with different concentrations of MG (0, 10, 30, 50, 100, 200, and 300 µM) for 48 h. It was observed that MG inhibited cell growth in a dose-dependent manner. Compared with the control group, exposing cells to 40, 60, and 80 µM of MG for 48 h resulted in a significant reduction in the number of cells (Rasul et al., 2012). MG significantly suppressed the proliferation of SKOV3 and TOV21G cells in a dose-dependent (6.25, 12.5, 25, 50, and 100 μM) and time-dependent (48 and 72 h) manner (Chuang et al., 2011). The QBC939, SK-ChA-1, MZ-ChA-1, and RBE cells were treated with different concentrations of MG (20, 40, 80, and 160 μM) for 24, 48, and 72 h. The results demonstrated that MG significantly suppressed the proliferation of the above cell lines in a concentration- and time-dependent manner (Zhang FH et al., 2017).

Fujita *et al.* investigated the ability of MG and honokiol to inhibit UV-induced mutation in *Salmonella typhimurium* TAI02. The results suggested that both MG (5 μg/per plate) and honokiol (5 μg/per plate) could inhibit against UV-induced mutations by scavenging ·OH generated by UV irradiation. The relative mutagenic activities of MG and honokiol were 62 ± 1% and 62 ± 4%, respectively, while that of control was 100% (Fujita and Taira, 1994). MG significantly inhibited the mutagenicity induced by indirect mutagens but did not affect the direct mutagens. It strongly and competitively inhibited the activities of ethoxyresorcinol-*O*-demethylase and methoxyresorcinol-*O*-demethylase, indicating that it could inhibit indirect mutagen-induced mutations by suppressing the activities of CYP1A1 and CYP1A2 (Saito et al., 2006). The genotoxicity of *Magnolia* bark extract (MBE) was studied by Li *et al.*, which was composed of 94% MG and 1.5% honokiol. The results revealed that MBE was not genotoxic under the conditions of the *in vitro* bacterial reverse mutation test and *in vivo* micronucleus test and supported the safety of MBE for dietary consumption (Li et al., 2007).

In general, the abovementioned cytotoxicity is mostly related to the antitumor and antiangiogenic activities of MG. Additionally, studies have shown that MG not only has no mutagenic and genotoxic activity but also even has antimutagenic activity. In summary, MG was found to be fairly nontoxic.

## Bioavailability and Formulation

MG is a dimeric phenolic neolignan (Pulvirenti et al., 2017) with strong lipid solubility, and its absorption in the gastrointestinal tract is mainly through a lipid-like pathway (Niu et al., 2015). Hatorri *et al.* studied the absorption, metabolism, and excretion of MG through oral administration and intraperitoneal injection of [ring-^14^C] MG. The results showed that MG participated in enterohepatic circulation (Hattori et al., 1986). After oral administration of MG (50 mg kg^−1^), the MG sulfates and glucuronides were predominant in the bloodstream. And MG was mainly distributed in the liver, kidney, brain, lung, and heart; among these organs, the concentration of MG and MG glucuronides in the liver was the highest (Lin et al., 2011). Additionally, MG’s main metabolite excreted in bile was magnolol-2-*O*-glucuronide, and the main route of excretion of MG after oral or intraperitoneal injection was through the alimentary tract (Hattori et al., 1986). After 24 h of oral administration of [ring-^14^C] MG, the main fecal derivatives of oral MG in rats were MG and a series of free form metabolites, which accounted for more than 90% of the total dose; only 6% were glucuronic acid and sulfate (Hattori et al., 1986). The MG metabolites tetrahydromagnolol and *trans*-isomagnolol showed an increasing trend after repeated administration, indicating that their formation was related to the induction of metabolic enzymes in animal tissues and/or intestinal bacteria. It was mainly excreted through liver metabolism and renal excretion (Hattori et al., 1986). The absorption half-life, elimination half-life (T_1/2_), maximum concentration-time (T_max_), and maximum concentration (C_max_) of MG were 0.63 h, 2.33 h, 1.12 h, and 0.16 μg ml^−1^, respectively. The water solubility and gastrointestinal absorption of MG were poor, with the oral bioavailability of only 4.9% (Tsai et al., 1996), limiting its clinical use. The low bioavailability might be partly due to the high metabolism of the intestine and liver and the low solubility in gastric juice.

In recent years, the bioavailability of MG has been significantly improved by various formulations including solid dispersion (Ochiuz et al., 2016; Tang et al., 2016; Stefanache et al., 2017b; Stefanache et al., 2017a; Li et al., 2019), phospholipid complex (Liu et al., 2020), liposome (Chen, 2008; Chen, 2009; Shen et al., 2016), nanoparticles (Wang et al., 2011), emulsion (Sheng et al., 2014), mixed micelles (Shen H et al., 2018; Ding et al., 2018), β-cyclodextrin inclusion compound (Qiu et al., 2016), and Zr-based organometallic framework (Santos et al., 2020) (Table 3).

**TABLE 3:** Formulations of MG.

Carrier	Proportion			Drug loading (%)	Entrapment efficiency (%)	Solubility (mg ml^−1^)	Bioavailability	References
Pluronic F127 and L61 (8:1; MG-M)	—			27.58 ± 0.53	81.57 ± 1.49	3.62 ± 0.02	The C_max_, AUC_0-∞_, T_max_, and T_1/2_ values of MG-M were 0.823 mg ml^−1^, 4.673 ± 0.31 mg/ml h, 0.75 ± 0.158 h, and 2.982 ± 0.528 h, respectively. The relative bioavailability of MG-M was 283% greater than that of raw MG.	Shen H et al. (2018)
SOL: HS15 40:10	—			4.12 ± 0.16	98.37 ± 1.23	—	The C_max_, AUC_0-∞_, T_max_, and T_1/2_ values of MG-H were 0.837 ± 0.050 μg ml^−1^, 5.127 ± 0.988 μg/ml h, 0.708 ± 0.188 h, and 3.656 ± 1.212 h, respectively. The relative oral bioavailability of MG-H increased by 2.98-fold.	Ding et al. (2018)
SOL: TPGS 50: 6	—			4.03 ± 0.19	94.61 ± 0.91	—	The C_max_, AUC_0-∞_, T_max_, and T_1/2_ values of MG-T were 0.918 ± 0.040 μg ml^−1^, 6.027 ± 0.963 mg/ml h, 0.750 ± 0.158 h, and 3.407 ± 0.855 h, respectively. The relative oral bioavailability of MG-T increased by 2.39-fold.	Ding et al. (2018)
Phospholipids, cholesterol, and mPEG2000-DSPE	Phospholipids: cholesterol: mPEG 2000-DSPE: MG 60:8:3:20			—	98.22	—	Compared with MG solution, the liposome had a sustained-release effect.	Shen et al. (2016)
Soy lecithin	Soy lecithin: MG 0.27:0.8			—	—	—	The cumulative dissolution rate was 96.3%, in 12 h. And the bioavailability was increased by 1.38 times, with the value of C_max_ for 533.62 ± 59.01 ng ml^−1^.	Liu et al. (2020)
PVP K30	PVPK30:MG 0.27:1.35			—	—	—	The cumulative dissolution rate was 76.4%, in 12 h. And the bioavailability was increased by 2.12 times, with the value of C_max_ for 721.73 ± 103.44 ng ml^−1^.
Povidone S-630 (PS-630)	PSS-630: MG 6:1			—	—	—	The value of relative bioavailability, AUC_0-t_, T_1/2_, and C_max_ was 137.22%, 823.81 ± 152.63 ng/L h, 6.066 ± 1.879 h, and 304.59 ± 136.48 ng L^−1^.	Li et al. (2019)
HPC	HPC: MG 6:1			—	—	—	The values of bioavailability, AUC_0-t_, T_1/2_, and C_max_ were 170.88%, 1025.90 ± 149.93 ng/L h, 17.63 ± 5.020 h, and 151.75 ± 26.37 ng L^−1^, respectively.	
Eudragit EPO (EPO)	EPO: MG 6:1			—	—	—	The values of bioavailability, AUC_0-t_, T_1/2_, and C_max_ were 79.50%, 477.30 ± 159.46 ng/L h, 13.81 ± 11.780 h, and 83.49 ± 22.37 ng L^−1^, respectively.	Lin et al. (2014)
EPC and DPPC	0.075 mg mL^−1^ MG			—	74.13 ± 1.97 (EPC), 64.26 ± 2.92	—	The EPC and DPPC liposomes enhanced the activity of inhibiting VSMC.	Chen (2008)
PVP	PVP: MG 1:1		—		—	105	The C_max_, AUC_0-∞_, and T_max_, values of solid dispersion were 0.6 ± 0.1 nmol ml^−1^, 679.0 ± 130.0 nmol/ml^−1^ min, and 275.0 ± 272.6 min, respectively.	Lin et al. (2014)
CHC	MG concentration from 0.05 to 0.2 mg ml^−1^		79.3 ± 2.2 (0.2 mg ml^−1^), 88.4 ± 2.3 (0.2 mg ml^−1^), and 91.6 ± 0.4 (0.2 mg ml^−1^)		—	—	Compared with free MG, MG-CHC nanoparticles showed better cell uptake efficiency, antiproliferation, and inhibition of VSMC migration.	Wang et al. (2011)
Oil phase mass fraction of 20 wt% and an aqueous phase mass fraction of 80 wt%	The amount of MG was 2.0 g/100 ml		—		—	—	The absolute bioavailability of MG is 17.5 ± 9.7%. The AUC_0-∞_, T_1/2_, CL/F, and Vd/F values of MG emulsion (25 mg kg^−1^, i.v.) were 6,875 ± 1,080 μg/ml h, 5.49 ± 1.77 h, 2.9 ± 0.9 ml/h/kg, and 0.37 ± 0.059 ml/kg, respectively. The C_max_, AUC_0-∞_, T_max_, T_1/2_, CL/F, and Vd/F values of MG emulsion (50 mg kg^−1^, oral administration) were 426.4 ± 273.8 ng ml^−1^, 2665 ± 1,306 μg/ml h, 1.2 ± 1.6 h, 4.9 ± 3.0 h, 2.2 ± 1.0 ml/h/kg, and 13.9 ± 5.1 ml kg^−1^, respectively.	Sheng et al. (2014)
						
Distearoyl phosphatidylcholine (DSPC), DPPC, and dimyristoyl phosphatidylcholine (DMPC)	—			—	84.87 ± 1.97 (DSPC), 75.05 ± 3.93 (DPPC), and 67.19 ± 2.92 (DMPC)	—	The three kinds of lipid could increase the inhibition activity of MG to VSMC, and the efficacy of inhibition was DMPC > DPPC > DSPC.	Chen (2009)
HP-β-CD	HP-β-CD: MG 10:1			—	—	—	The water solubility of HP-β-CD-MG was more than 500 times higher than that of MG, and the stability of HP-β-CD-MG was significantly increased.	Qiu et al. (2016)
Uio-66(Zr)	—			—	72.16 ± 2.15	—	The C_max_, AUC_0-∞_, T_max_, and T_1/2_ values of MG@Uio-66(Zr) (100 mg kg^−1^, oral administration) were 3.77 ± 0.33 μg ml^−1^, 2099.95 ± 148.48 μg/ml min, 196.97 ± 17.38 min, and 206.21 ± 27.95 min, respectively. The C_max_, AUC_0-∞_, T_max_, and T_1/2_ values of MG@Uio-66(Zr) (100 mg kg^−1^, i.p.) were 5.65 ± 2.41 μg ml^−1^, 3831.72 ± 451.57 μg/ml min, 114.27 ± 7.09 min, and 606.35 ± 114.37 min, respectively. The relative bioavailability increased almost two-fold.	Santos et al. (2020)
						
Soluplus VR and Poloxamer 188		MG: Soluplus VR: Poloxamer 188 1:12:5(MG-loaded mixed micelles (MMs)) and 2:1:1(MG nanosuspensions (MNs))	5.46 ± 0.65% (MMs) and 42.50 ± 1.57% (MNs)		89.58 ± 2.54% (MMs)	—	The C_max_, AUC_0-∞_, T_max_, and T_1/2_ values of MMs were 0.587 ± 0.048 mg L^−1^, 2.904 ± 0.465 μg/L h, 0.792 ± 0.102 h, and 3.142 ± 0.285 h, respectively. The C_max_, AUC_0-∞_, T_max_, and T_1/2_ values of MNs were 0.65 ± 0.125 mg L^−1^, 2 2.217 ± 0.332 μg/L h, 0.5 h, and 2.776 ± 0.417 h, respectively. The gastrointestinal absorption of MG was increased by 2.85 and 2.27 times by MM and MN, respectively.	Li G et al. (2020)
PVP K-30		MG: PVP K-30 1:1	—		—	—	The C_max_, AUC_0-∞_, and T_max_ values of solid dispersion were 0.6 ± 0.1 nmol ml^−1^, 679.0 ± 130.0 nmol/min mL, and 275.0 ± 272.6 min, respectively.	Lin et al. (2014)

Liu *et al.* prepared MG solid dispersion, MG solid lipid nanoparticles, and MG phospholipid complex and studied their bioavailability. The results showed that the cumulative dissolution of MG was 30.6% within 12 h, while the cumulative dissolution of MG solid dispersion, MG solid lipid nanoparticles, and MG phospholipid complex increased to 96.3, 76.4, and 45.9%, respectively. The pharmacokinetic parameters such as C_max_ and area under the curve (AUC)_0-t_ and AUC_0-∞_ were significantly improved. Moreover, compared with raw MG, their relative bioavailability increased to 1.38, 2.12, and 3.45 times, respectively (Liu et al., 2020). All three preparations could improve the oral absorption bioavailability of MG, but the effect of MG solid lipid nanoparticles was more obvious. Lin *et al.* prepared a solid dispersion of MG with polyvinylpyrrolidone K-30 (PVP) and studied its bioavailability by oral administration (50 mg kg^−1^). The results indicated that compared with raw MG, the solid dispersion of MG with PVP significantly increased the systemic exposures of MG and MG sulfates/glucuronides by 80.1 and 142.8%, respectively (Lin et al., 2014). For the solid dispersion prepared by MG and croscarmellose sodium (1: 5), the *in vitro* cumulative dissolution rate of MG reached 80.66% at 120 min, which was 6.9 times that of the raw MG (11.74%) (Tang et al., 2016). Stefanache *et al.* incorporated MG into the pores of amino-functionalized mesoporous silica particles to increase the dosage of MG and delay its release (Stefanache et al., 2017a).

After gavaging the emulsion (50 mg kg^−1^) in male SD rats, the 1.20 h average plasma concentration of MG was 426.4727 ng ml^−1^, and the absolute bioavailability was 17.579%, indicating that preparing an emulsion could improve the bioavailability of MG (Sheng et al., 2014).

Chen used 1,2-diacyl-Sn-glycero-3-phosphocholine (EPC) and 1,2-dipalmitoyl- Sn-glycero-3-phosphocholine (DPPC) liposomes to encapsulate MG with entrapment efficiencies of 74.13 ± 1.97% and 64.26 ± 2.92%, respectively. The results showed that EPC and DPPC liposomes enhanced the inhibitory effect of MG on VSMCs, and the inhibitory effect of EPC liposome-encapsulated MG on VSMCs was better than that of DPPC liposome (Chen, 2008). Qiu *et al.* utilized hydroxypropyl-β-cyclodextrin (HP-β-CD) to prepare MG-HP-β-CD inclusion complex (HP-β-CD: MG 10:1). The stoichiometric ratio of the inclusion compound was 1:1, and the stability constant (Kc) was calculated as 2206 M^−1^. In addition, the aqueous solubility of the MG-HP-β-CD inclusion complex was more than 500-fold that of free MG, and it had better stability and stronger antitumor activity *in vitro* (Qiu et al., 2016). Santos *et al.* used Uio-66 (Zr) as the MG carrier. After oral or intraperitoneal administration of 100 mg kg^−1^ of MG and MG@Uio-66(Zr), the AUC_0-720_ of MG@Uio-66(Zr) (op:1823 ± 167.31 μg/ml min, i.p.: 2312.67 ± 253.76 μg/ml min) was significantly higher than the AUC_0-720_ of free MG (op: 823.3 ± 139.10 μg/ml min, i.p.: 2582.67 ± 150.48 μg/ml min). The relative bioavailability of MG increased almost twofold by using Uio-66(Zr) (Santos et al., 2020). The drug loading efficiency of MG-carboxymethyl-hexanoyl chitosan (CHC) nanoparticles was in the range of 91.6 ± 0.4 to 79.3 ± 2.2%, depending on the initial MG concentration of 0.05–0.2 mg mL^−1^. MG**-**CHC nanoparticles had excellent cell uptake efficiency. Compared with free MG, it could be effectively delivered within the cell, which increased the resistance proliferation and inhibition of VSMC migration (Wang et al., 2011).

Mixed Soluplus (SOL) and Solutol HS15 (HS15), SOL, and D-alpha-tocopheryl polyethylene glycol 1,000 succinate (TPGS) were used to prepare MG-loaded mixed micelles (MG-M) MG-H and MG-T, respectively. The relative oral bioavailability of MG-T and MG-H were increased by 2.39- and 2.98-fold, respectively, compared to that of raw MG, indicating that MG-H and MG-T could promote the absorption of MG in the gastrointestinal tract (Ding et al., 2018). Shen *et al.* also prepared MG-M by pluronic F127 and L61, and its drug loading efficiency and entrapment efficiency were 81.57 ± 1.49% and 27.58 ± 0.53%, respectively. *In vitro* release test showed that MG had sustained release behavior after being encapsulated in micelles. The permeability of MG through the Caco-2 cell monolayer was enhanced, and the relative bioavailability of oral MG-M was 2.83 times higher than that of the raw MG (Shen H et al., 2018). It can be seen that the mixed micelle drug delivery system can improve the poor water solubility and bioavailability of MG.

In general, the existing formulations can not only improve the water solubility and bioavailability of MG but also improve its stability, enhance its pharmacological effects, and enable MG to have a sustained release behavior, which will provide strategies for future clinical applications of MG.

## Conclusion

In 2011, Chen *et al.* summarized the pharmacological activities and molecular mechanisms of MG. According to the review, MG could exhibit anti-inflammatory activity by inhibiting the production of inflammatory enzymes/cytokines and activation of NF-κB and leukocyte. It also exerted antitumor effects by inhibiting cell proliferation and metastasis and inducing apoptosis. The molecular mechanisms mainly include the increase of p21, p27, caspase-3, caspase-8, and caspase-9 expression, inhibition of PI3K/PTEN/AKT pathway, ERK1/2, NF-κB, P38, iNOS, and COX2 activation, CYP1A1, CYP1A2, MMP-9 as well as MMP-2 activity and Bcl-2 expression, induction of cytochrome C, and AIF release and activation of the mitochondrial death receptor pathway. MG could attenuate VCAM-1, ICAM-1, MCP-1, and MMP9, inhibit the proliferation of smooth muscle cells and fibroblasts, and obtain arrhythmia from I/R injury to show cardiovascular protection. It could also exert neuroprotective activities by inhibiting the production of PGE2, regulating (GABA)_A_ receptor subtypes and central serotonergic activity, retaining cholinergic neurons in the forebrain, and inhibiting cortical 5-HT release. MG had a therapeutic effect on gastrointestinal diseases by regulating serotonergic and gastrointestinal system functions and relaxing gastrointestinal smooth muscles. Moreover, it exhibited hypoglycemic activity by activating PPAR and increasing basal and insulin-stimulated glucose uptake (Chen YH et al., 2011).

In this review, *in vivo* and *in vitro* studies demonstrated that MG has a wide range of pharmacological activities including anti-inflammatory, antitumor, antioxidant, hypoglycemic, cardiovascular protection, antiangiogenesis, and antibacterial. MG inhibited TLR2/TLR4/NF-κB/MAPK/PPAR-γ pathways and decreased the expression of inflammatory cytokines to exhibit anti-inflammatory activity. It suppressed the growth, migration, and invasion of tumor cells and promoted apoptosis as well as autophagy, through acting on caspase-8, caspase-3, and other proteins participated in the p53, MAPK, NF-κB, TLR, PI3K/Akt/mTOR, and Wnt/β-catenin signaling pathways. It also protected the nervous system through multiple systems and multiple targets. Moreover, it has a wide range of antibacterial activity. MG is a candidate drug for anti-inflammatory, anticancer, and neuroprotective activities. However, MG’s *in vivo* effect with CYP enzymes is not clear yet, and there is no clinical research on MG, which cannot fully provide the pharmacological activities of it.

MG and honokiol have similar pharmacological activities. Both of them can exhibit antitumor activities by regulating MAPK, NF-κB, HIF-α, PI3K/Akt/ERK/mTOR, and Wnt/β-catenin signaling pathways. MG shows antitumor activity by regulating TLR signaling pathways, and honokiol can regulate STAF, EGFR, and notch signaling pathways to exhibit antitumor activities. They have inhibitory activity on α-glucosidase and stimulation of glucose uptake to play a hypoglycemic role, while MG has a better inhibitory effect of α-glucosidase. Moreover, both MG and honokiol exhibit gastrointestinal protective activity with similar mechanism, while MG’s antidiarrheal activity is better than that of honokiol. MG is a partial agonist of CB1 and CB2; however, honokiol is a full agonist of CB1 and an inverse agonist of CB2. MG has no activity on GPR-55, while honokiol is an antagonist of GPR-55. Honokiol has a stronger positive regulatory effect on GABAA receptors than MG; however, MG is more effective in enhancing PPAR-γ luciferase levels than honokiol. What is more, the inhibition types of MG on CYP1A, CYP2C19, CYP2C, CYP3A, and CYP1A2 were competitive inhibition. The inhibition type of honokiol on CYP1A2 was competitive inhibition, and the inhibition types of honokiol on CYP2E1 and CYP2C19 were noncompetitive inhibition. Both honokiol and MG have antimicrobial activity. The difference is that honokiol exhibits better antimicrobial activity than MG on *Aggregatibacter actinomycetemcomitans*, *S. mutans*, *S. aureus*, MRSA, *Escherichia coli*, and *Fusarium* spp.

MG is nontoxic and is used in dietary supplements and cosmetic products, such as added to toothpaste to play antibacterial and antiperiodontitis effects. However, the low water solubility, poor bioavailability, and skin irritation hamper its application. To overcome this problem, numerous studies have been conducted. By preparing solid dispersions, nanoparticles, phospholipid complexes, liposomes, emulsions, *etc*., the bioavailability and stability of MG significantly improved, which will greatly promote its clinical application. Aside from its formulations, structural modification is becoming an increasingly promising method for obtaining MG derivatives with better therapeutic effects and higher bioavailability. The synthesis and research of MG derivatives are beyond the scope of this study, so we will not go into details. Consequently, the design and research of MG derivatives are of great significance in the future.

In summary, this article comprehensively reviews the pharmacology, toxicity, bioavailability, and formulations of MG.

## Glossary

(iNOS): inducible nitric oxide synthase
(COX-2): cyclooxygenase-2
(NF-κB): nuclear factor-κB
(MAPK): mitogen-activated protein kinase
(PGEs): prostaglandins
(ERK): extracellular signal-regulated kinase
(BMK1): big mitogen-activated protein kinase 1
(SAPK): stress-activated protein kinase
(JNK): c-Jun N-terminal kinase
(NO): nitric oxide: nitric oxide
(TLR): Toll-like receptor
(PI3K): phosphatidylinositol 3-kinase
(Akt): Protein kinase B
(LPS): lipopolysaccharide
(IκB): inhibitor kappa B: inhibitor κB
(Nrf2): nuclear factor-erythroid 2-related factor 2
(HO): heme oxygenase
(SD): Sprague Dawley
(PPAR-γ): peroxisome proliferator-activated receptor-γ
(ROS): reactive oxygen species
(MMP): matrix metalloproteinase
(PCNA): proliferating cell nuclear antigen
(CCA): cholangiocarcinoma
(Bcl-2): B-cell lymphoma-2
(mTOR): mammalian target of rapamycin
(PKC): protein kinase C
(VSMC): vascular smooth muscle cell
(NO): nitric oxide: nitric oxide
(cGMP): guanosine 3′,5′-cyclic phosphate
(ACE): angiotensin-converting enzyme
(Ang II): angiotensin II
(AT-1R): Ang II type 1 receptor
(FAK): focal adhesion kinase
(MEK): mitogen-activated protein kinase
(eNOS): endothelial nitric oxide synthase
(MES): mouse embryonic stem
(EB): embryoid body
(VEGF): vascular endothelial growth factor
(HIF-1a): hypoxia-inducible factor-1a
(ET-1): endothelin-1
(TNF-α): tumor necrosis factor-α
(PAH): pulmonary arterial hypertension
(DSS): dextran sulfate sodium
(IL-1β): interleukin-1β
(IκB): inhibitor kappa B: inhibitor κB
(ICAM-1): intercellular adhesion molecule-1
(Cdk2): cyclin-dependent kinase 2
(IGF-1): insulin-like growth factor-1
(IGFBP-5): IGF-binding protein-5
(IGF-1R): IGF-1 receptor
(MPO): myeloperoxidase
(PKA): protein kinase A
(AD): Alzheimer’s disease
(CYP): cytochrome P450 monooxygenase
(SOD): superoxide dismutase
(GSH-Px): glutathione peroxidase
(CORT): corticosterone
(GSH): glutathione
(AA): aristolochic acid
(MIC): minimal inhibitory concentration
(MBC): minimal bactericidal concentration
(SIRT1): silent information regulator 1
(NE): norepinephrine
(BDNF): brain-derived neurotrophic factor
(HPA): hypothalamic–pituitary–adrenal
(5-HT): 5-hydroxytryptamine
(4-HNE): 4-hydroxynonenal
(FoxO): O subfamily of forkhead transcription factors
(HASMCs): human aortic smooth muscle cells
(Cdc42): cell division cycle 42
(GSK3β): glycogen synthase kinase 3β
(HEK): human embryonic kidney
(I-R): ischemia–reperfusion
(CMS): chronic mild stress
(BBB): blood–brain barrier
(LC50): lethal concentration
(CAT): catalase
(p-JNK): phospho-JNK
(MRSA): methicillin-resistant *Staphylococcus aureus*
(MSSA): methicillin-susceptible *S. aureus*
(CDC): cell division cycle gene
(PS-630): Povidone S-630
(HPC): hydroxypropyl cellulose
(EPO): Eudragit EPO
(PVP K30): polyvinylpyrrolidone K30
(CMC): carboxymethylcellulose: croscarmellose sodium
(BMP2): bone morphogenetic protein 2
(RUNX2): runt-related transcription factor 2
(ApoE): apolipoprotein E
(i.v.): intravenous
(i.p.): intraperitoneal
(CMC): carboxymethylcellulose: croscarmellose sodium
(AUC): area under the curve
(T_1/2_): elimination half-life
(T_max_): maximum concentration-time
(C_max_): maximum concentration
(GBC): gallbladder carcinoma
(TMT): trimethyltin
